# Glycosylation Significantly Inhibits the Aggregation of Human Prion Protein and Decreases Its Cytotoxicity

**DOI:** 10.1038/s41598-018-30770-6

**Published:** 2018-08-22

**Authors:** Chuan-Wei Yi, Li-Qiang Wang, Jun-Jie Huang, Kai Pan, Jie Chen, Yi Liang

**Affiliations:** State Key Laboratory of Virology, College of Life Sciences, Wuhan University, Wuhan, 430072 China

## Abstract

Prion diseases are primarily caused by the misfolding of prion proteins in humans, cattle, sheep, and cervid species. The effects of glycosylation on prion protein (PrP) structure and function have not been thoroughly elucidated to date. In this study, we attempt to elucidate the effects of glycosylation on the aggregation and toxicity of human PrP. As revealed by immunocytochemical staining, wild-type PrP and its monoglycosylated mutants N181D, N197D, and T199N/N181D/N197D are primarily attached to the plasma membrane. In contrast, PrP F198S, a pathological mutant with an altered residue within the glycosylation site, and an unglycosylated PrP mutant, N181D/N197D, primarily exist in the cytoplasm. In the pathological mutant V180I, there is an equal mix of membranous and cytoplasmic PrP, indicating that N-linked glycosylation deficiency impairs the correct localization of human PrP at the plasma membrane. As shown by immunoblotting and flow cytometry, human PrP located in the cytoplasm displays considerably greater PK resistance and aggregation ability and is associated with considerably higher cellular ROS levels than PrP located on the plasma membrane. Furthermore, glycosylation deficiency enhances human PrP cytotoxicity induced by MG132 or the toxic prion peptide PrP 106-126. Therefore, we propose that glycosylation acts as a necessary cofactor in determining PrP localization on the plasma membrane and that it significantly inhibits the aggregation of human PrP and decreases its cytotoxicity.

## Introduction

Prions were first described by the Nobel laureate Stanley B. Prusiner. The word “prion” is derived from the words “proteinaceous” and “infectious” and means “protein infectious agent”^1^. Prion diseases, which are formally called transmissible spongiform encephalopathies (TSEs), are primarily caused by the misfolding of prion proteins in humans, cattle, sheep, and cervid species^2–5^. Moreover, the concept of prion diseases has been extended to prion-like diseases^6^, examples of which are Parkinson’s disease caused by the misfolding of α-synuclein, Alzheimer’s disease caused by the misfolding of Tau protein and amyloid β peptide, and amyotrophic lateral sclerosis caused by the misfolding of SOD1 and TDP-43^6–9^. The normally folded, cellular form of the prion protein, PrP^C^, has important functions in peripheral myelin maintenance, memory formation, neuroprotection, immune system activation, circadian rhythm, and metal ion homeostasis^10–14^. However, misfolded PrP^C^ can be transformed into the pathologic form, PrP^Sc^, which is the major component of the infectious agent found in prion diseases^1,15^. Although the primary structures of PrP^C^ and PrP^Sc^ are the same, there are many differences in their three-dimensional structures, physicochemical properties, and functions, including β-sheet content, detergent insolubility, and resistance to proteinase K (PK) activity^1,2,16^.

Human PrP^C^ is a glycoprotein that is tethered to the plasma membrane *via* a glycosylphosphatidylinositol (GPI) anchor^17^. The full-length human prion protein (PrP) is composed of 254 amino acid residues and two signal peptides. The N-terminal signal peptide guides the precursor PrP into the endoplasmic reticulum (ER) for further processing; mature PrP then reaches the plasma membrane with the help of the C-terminal signal peptide and the GPI anchor^17–19^. Human PrP has two glycosylation sites, Asn-181 and Asn-197, in its C-terminal^17,20–22^ region. Sialylation, a terminal modification involving glycans, has been reported^23^. Each glycan can connect covalently with two or more sialic acids, and sialylation changes the isoelectric point of PrP due to electrostatic interactions^23–25^. Sialylation can regulate PrP-mediated cell signaling, control prion replication rate and glycoform ratio, and influence prion strains and infectivity^24–31^. The N-linked glycosylation of PrP has been widely studied in the past 30 years, and always occurs at the sequence AsnXaaThr or AsnXaaSer^32^. Glycosylation is very flexible and occupies a large part of the PrP surface, and there are more than 50 monosaccharides in the saccharide groups of PrP^33^. Capellari and colleagues have reported that the formation of disulfide bonds changes the glycosylation state and increases the formation of unglycosylated PrP^34^. It has been demonstrated that glycosylation of PrP stabilizes its secondary structure and prevents it from adopting a β-sheet conformation^20,21,35–39^. Although a defined stoichiometry of PrP^Sc^ glycoforms and of PrP^C^ glycoforms is dispensable for efficient conversion by protein misfolding cyclic amplification^40^ and glycosylation is not necessary for the acquisition of protease resistance by PrP in scrapie-infected cells^41^, glycosylation determines prion replication, strains, cytotoxicity and neuroinvasion^42–44^. In reviewing previous studies, we found that in many pathological mutants of human PrP, including D178N, V180I, T183A, F198S, E196K, and E200K, the residues near the glocosylation sites are altered^3,5^. The Prusiner laboratory has expressed Syrian hamster PrP in the CV1 cell line and shown that both single glycosylation site mutants are mono glycosylated and located in the cytoplasm and that the double mutant T183A/T199A is unglycosylated and is also located in the cytoplasm, while wild-type PrP is localized to the plasma membrane^45^. Cancellotti and colleagues have described transgenic mouse models of glycosylation and found that the double mutant N180T/N196T mainly exists in the cytoplasm, whereas wild-type PrP and its two single glycosylation site mutants are localized to the plasma membrane^46^. Salamat and coworkers expressed ovine PrP in RK13 cells and found that two single glycosylation site mutants are located partially in the Golgi apparatus but mainly on the plasma membrane, while the double mutant N184D/N200D is located in the cytoplasm^47^. Lehmann and Harris expressed mouse PrP in Chinese hamster ovary cells and found that the mono glycosylated mutant T182A and the unglycosylated mutant T182A/T198A exist in the cytoplasm whereas wild-type PrP and another mono glycosylated mutant, T198A, are localized to the plasma membrane^48^. The Kretzschmar laboratory expressed mouse PrP in N2a cells and found that the mono glycosylated mutant T182A exists in the cytoplasm whereas wild-type PrP is localized to the plasma membrane^49^. However, Gao and colleagues investigated the expression of human PrP with other substitution mutations (N181A/N197A) in Chinese hamster ovary cells and reported that PrP glycosylation is not essential for the expression of PrP on the cell surface^50^ because PrP is a GPI-anchored membrane protein^51^. Thus, existing information on the glycosylation, subcellular location, and aggregation of PrP is controversial and complex, and it is possible that additional data could clarify some of these issues.

In this study, we first investigated whether glycosylation alters the subcellular location of human PrP and found that deficient N-linked glycosylation impaired the correct localization of human PrP on the plasma membrane. Secondly, we studied whether the change in the subcellular location of PrP altered its aggregation or influenced PrP aggregation-induced cytotoxicity. Our results demonstrated that human PrP with a lower degree of glycosylation has higher PK resistance, greater aggregation ability, is associated with higher intracellular levels of reactive oxygen species (ROS), and induces a higher percentage of apoptosis than PrP with a higher degree of glycosylation. We concluded that glycosylation significantly inhibits the aggregation of human PrP and decreases its cytotoxicity.

## Results

### N-linked glycosylation deficiency impairs the correct localization of human PrP at the plasma membrane

**Figure 1:**
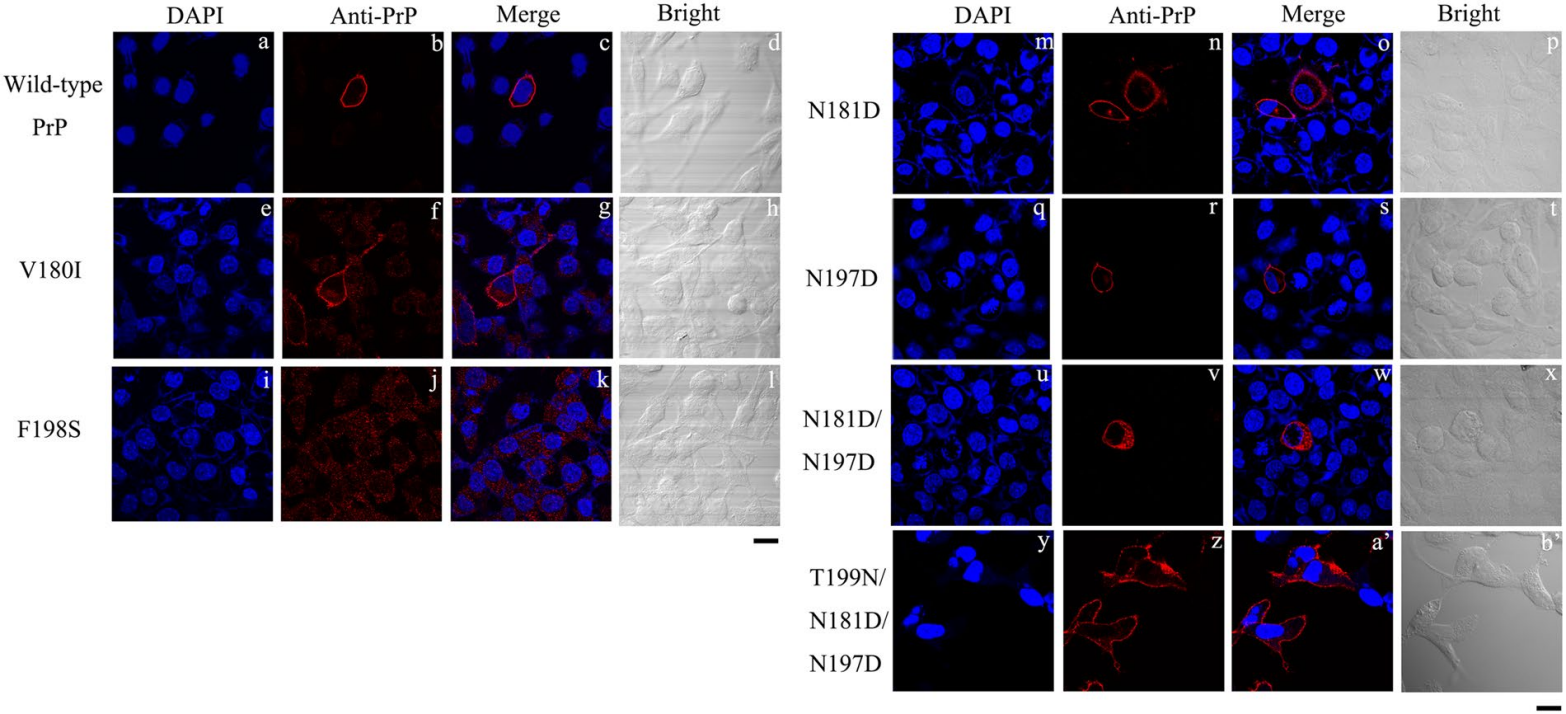
N-linked glycosylation deficiency impairs the correct localization of human PrP at the plasma membrane of RK13 cells. RK13 cells transiently expressing wild-type PrP **(a–d)**, V180I **(e–h)**, F198S **(i–l)**, N181D **(m–p)**, N197D **(q–t)**, N181D/N197D **(u–x)**, and T199N/N181D/N197D **(y–b)** were cultured for 2 days at 37 °C, fixed, permeabilized, immunostained with the anti-PrP antibody 3F4 and with IgG conjugated to Alexa Fluor 546 (red), stained with DAPI (blue), and observed by confocal microscopy. The scale bars represent 10 μm.

Although glycosylation ratios contribute to the diversity of prion strains^22,24,26,47^, little is known about how glycosylation affects the biochemical properties and aggregation of PrP. We therefore investigated the effect of N-linked glycosylation on the subcellular localization of human PrP. These studies utilized the two pathological PrP mutants V180I and F198S, the three monoglycosylated mutants N181D, N197D, and T199N/N181D/N197D, and the unglycosylated mutant N181D/N197D. RK13 cells transiently (Fig. 1) or stably (Fig. S1) overexpressing wild-type or mutant PrP and SH-SY5Y cells transiently (Fig. S2) overexpressing wild-type or mutant PrP were plated on coverslips, incubated in medium for 48 h, and then harvested. We chose the RK13 cell line, a rabbit kidney epithelial cell line, and the SH-SY5Y cell line, a human neuroblastoma cell line, because the former does not endogenously express PrP and the latter has very low endogenous PrP expression, reducing the likelihood of false positive results. As shown in the representative images in Figs 1, S1, and S2, the subcellular location of wild-type PrP (a-d), V180I (e–h), F198S (i–l), N181D (m–p), N197D (q–t), N181D/N197D (u–x), and T199N/N181D/N197D (y–b′) in RK13 and SH-SY5Y cells was determined by confocal microscopy using the anti-PrP antibody 3F4 (red) and DAPI (blue), a nuclear stain. As revealed by the immunocytochemical staining, wild-type PrP and its monoglycosylated mutants, N181D, N197D, and T199N/N181D/N197D, were mainly attached to the plasma membrane (Fig. 1a–d, S1a–d, S2a–d, 1m–t, S1m–t, S2m–t, and 1y–b′). In contrast, the pathological mutant F198S, in which one of the glycosylation sites of PrP has been altered, and the unglycosylated PrP mutant N181D/N197D appeared mainly in the cytoplasm (Figs 1i–l, S1i–l, S2i–l, 1u–x, S1u–x, and S2u–x). In the case of the pathological mutant V180I, an equal mixture of membranous and cytoplasmic PrP was observed (Figs 1e–h, S1e–h, and S2e–h). These observations indicate that N-linked glycosylation deficiency impairs the correct localization of human PrP on the plasma membrane of RK13 and SH-SY5Y cells. PrP localization in stably expressing cells (Fig. S1) is clearly similar to that in transiently transfected cells (Fig. 1), although only a highly vacuolated N181D/N197D positive cell is shown in Fig. 1.

**Figure 2:**
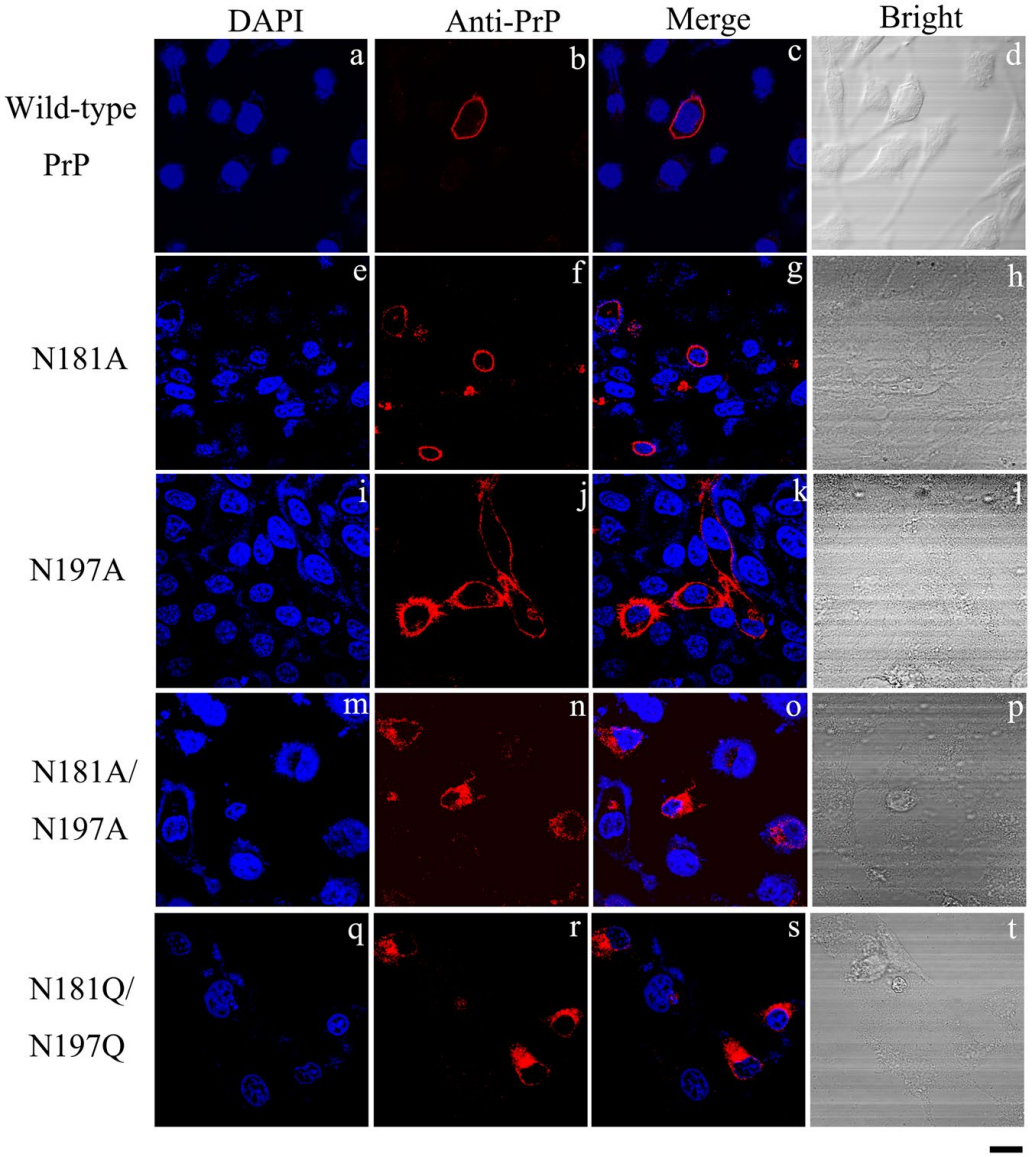
N-linked glycosylation deficiency impairs the correct localization of human PrP at the plasma membrane of RK13 cells. RK13 cells transiently expressing wild-type PrP **(a–d)**, N181A **(e–h)**, N197A **(i–l)**, N181A/N197A **(m–p)**, and N181Q/N197Q **(q–t)** were cultured for 2 days at 37 °C, fixed, permeabilized, immunostained with the anti-PrP antibody 3F4 and with IgG conjugated to Alexa Fluor 546 (red), stained with DAPI (blue), and observed by confocal microscopy. The scale bars represent 10 μm.

Without comparing the effects of different substitutions that alter the glycosylation sites of PrP, it is difficult to determine whether the deglycosylation of PrP or the introduction of a double mutation produced the observed effects on the protein and on the cells. Therefore, we tested the N181A/N197A and N181Q/N197Q mutants in RK13 cells (Fig. 2) and SH-SY5Y cells (Fig. S3). As revealed by immunocytochemical staining, wild-type PrP and its monoglycosylated mutants N181A and N197A were mainly attached to the plasma membrane (Figs 2a–l and S3a–l). In contrast, the two unglycosylated mutants of PrP, N181A/N197A and N181Q/N197Q, were present mainly in the cytoplasm (Figs 2m–t and S3m–t), once again indicating that N-linked glycosylation deficiency impairs the correct localization of human PrP at the plasma membrane of RK13 and SH-SY5Y cells.

**Figure 3:**
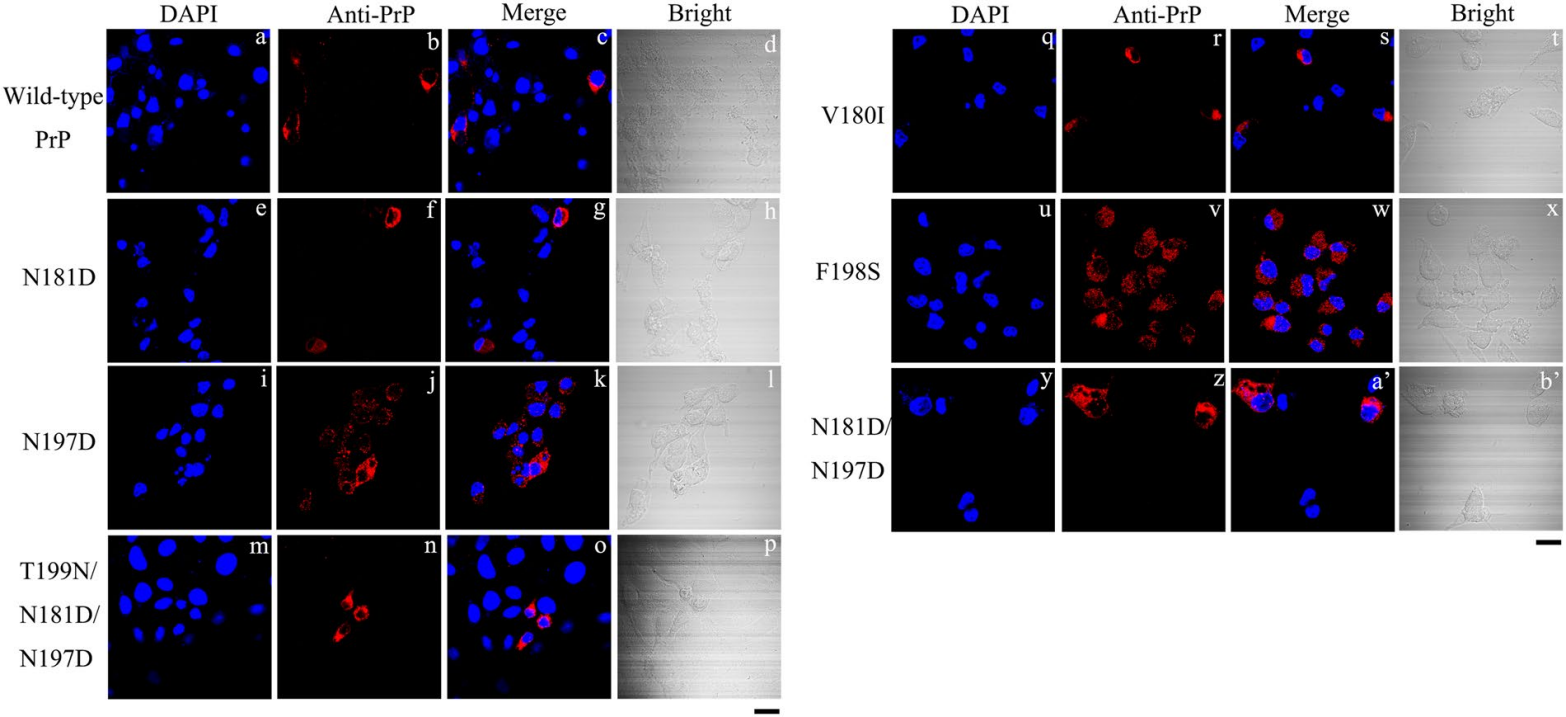
Tunicamycin suppresses glycosylation and results in the translocation of human PrP from the plasma membrane to the cytoplasm of RK13 cells. RK13 cells transiently expressing wild-type PrP **(a–d)**, N181D **(e–h)**, N197D **(i–l)**, T199N/N181D/N197D **(m–p)**, V180I **(q-t)**, F198S **(u-x)**, and N181D/N197D **(y-b’)** were cultured for 2 days and treated with 5 μg/ml tunicamycin for 36 h at 37 °C, fixed, permeabilized, immunostained with the anti-PrP antibody 3F4 and with IgG conjugated to Alexa Fluor 546 (red), stained with DAPI (blue), and observed by confocal microscopy. The scale bars represent 10 μm.

The above results suggest that impaired trafficking of mutant human PrP depends neither on the cell line nor on the specific amino acids chosen for the substitutions. Tunicamycin acts as an inhibitor of glycosylation by inhibiting the activity of GlcNAc phosphotransferase^52^. We investigated the effect of tunicamycin on the subcellular location of human PrP in RK13 cells. RK13 cells transiently overexpressing human PrP were treated with 5 ng/μl tunicamycin for 36 h. The subcellular locations of wild-type PrP and of the six mutant PrP proteins were then detected by confocal microscopy using an anti-PrP antibody 3F4 (red) and DAPI (blue) (Fig. 3). As shown in Fig. 3, human PrP was presently mainly in the cytoplasm after inhibition of N-linked glycosylation by tunicamycin. This was especially true for wild-type PrP and its monoglycosylated mutants N181D, N197D, and T199N/N181D/N197D (Fig. 3a–p); in the absence of tunicamycin treatment, all of the proteins were primarily located at the plasma membrane (Figs 1a–d,m–t, and y–b′). However, the tunicamycin experiments should be interpreted with caution because tunicamycin affects the N-linked glycosylation of every glycoprotein produced in the cells. Clearly, glycosylation at Asn-181 and Asn-197 is necessary for human PrP localization at the plasma membrane.

### Human PrP located in the cytoplasm has much stronger PK resistance and greater aggregation ability and induces much higher intracellular ROS levels than PrP located on the plasma membrane

**Figure 4:**
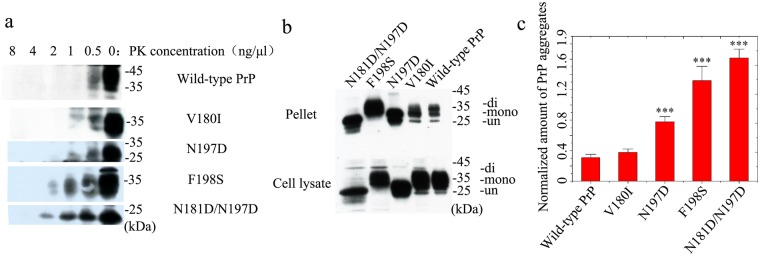
Human PrP located in the cytoplasm has much greater PK resistance and aggregation ability than PrP located on the plasma membrane of RK13 cells. RK13 cells stably expressing wild-type PrP, V180I, N197D, F198S, or N181D/N197D were cultured for 7 days. Wild-type PrP and its mutants in RK13 cells were digested with various concentrations of PK (from right to left, 0, 0.5, 1.0, 2.0, 4.0, and 8.0 ng/μl) and probed using the anti-PrP antibody 3F4 **(a)**. The normalized amount of insoluble PrP aggregates in the stable cells **(c)** was calculated as the ratio of the density of insoluble PrP aggregate bands probed by 3F4 to that of the total PrP bands in cell lysates also probed by 3F4 **(b)**. RK13 cells stably expressing wild-type PrP were used as a control. The diglycosylated, monoglycosylated, and unglycosylated bands are referred to as “di-”, “mono-”, and “un-”, respectively, and are annotated in **(b)**. Data on the normalized amounts of insoluble PrP aggregates are expressed as the mean ± S.D. (with error bars) of the values obtained in 3 independent experiments **(c)**. Statistical analyses were performed using Student’s *t*-test as described in the legend of Fig. 5. The images of wild-type PrP (or V180I) and N197D (or F198S or N181D/N197D) shown were obtained from two different gels using the same exposure time (30 s) **(a)**. The gels and blots of wild-type PrP, V180I, N197D, F198S, and N181D/N197D **(b)** were cropped from different parts of the same gel with an exposure time of 30 s. Clear delineation with white spaces was used. Full-length blots and gels are presented in Supplementary Figure 5.

We suggest that the subcellular location of PrP might play an important role as a bridge between N-linked glycosylation and the aggregation of PrP. According to the nucleation-polymerization model^16,53^, PrP monomers in the cytoplasm have a much greater tendency to form aggregating seeds than PrP monomers that are attached to the plasma membrane. To distinguish PrP^Sc^ from PrP^C^, the Prusiner laboratory pioneered a study of PK resistance activity^54^. In the present work, PK resistance activity was measured to highlight differences between wild-type PrP and its mutants related to two glycosylation sites. RK13 cells stably expressing wild-type PrP, V180I, N197D, F198S, and N181D/N197D were cultured for 7 days. Cells expressing either wild-type or mutant PrP were digested with various concentrations of PK and probed using the anti-PrP antibody 3F4 (Fig. 4a). Full-length blots and gels are presented in Fig. S4a. As can be seen in Figs 4a and S4a, a 35–45-kDa band of wild-type PrP, a 35-kDa band of V180I, 25–35-kDa bands of N197D, a 25–35-kDa band of F198S, and a 25-kDa band of N181D/N197D were heavily immunostained in the absence of PK but showed only weak immunostaining in the presence of 0.5 ng/μl PK. These bands did not appear when the PK concentration was increased to 1 ng/μl for wild-type PrP, to 2 ng/μl for V180I and N197D, and to 4 ng/μl for F198S and N181D/N197D (Figs 4a and S4a). As shown in Fig. S4a, an additional unexpected and previously uncharacterized band of 16 kDa was present in cells expressing wild-type or mutant PrP; this band, which represents unglycosylated PrP, may result from N- or C-terminal truncation of the protein in the cells or in the lysate. After ultracentrifugation of the samples, the normalized amounts of insoluble PrP aggregates in cells (Fig. 4c) were calculated as the ratio of the density of insoluble PrP aggregate bands probed with 3F4 to that of the total PrP bands in cell lysates probed with 3F4 (Fig. 4b). Full-length blots and gels are presented in Fig. S4b. The diglycosylated, monoglycosylated, and unglycosylated bands are labeled “di-”, “mono-”, and “un-”, respectively (Figs 4b and S4b). The densities of the 25-kDa band of N181D/N197D, the 35-kDa band of F198S, and the 25–35-kDa bands of N197D in the pellets were much greater than those of the 35-kDa and 25-kDa bands of wild-type PrP. Clearly, N181D/N197D, F198S, and N197D formed significantly higher levels of sarcosyl-insoluble aggregates than wild-type PrP (1.61 ± 0.12 for N181D/N197D, 1.32 ± 0.19 for F198S, and 0.779 ± 0.067 for N197D versus 0.308 ± 0.042 for wild-type PrP; *p* = 0.000058, 0.00080, and 0.00051, respectively) (Fig. 4c). We thus concluded that unglycosylated N181D/N197D, which is mainly located in the cytoplasm, has the strongest PK resistance and aggregation ability, followed by the pathological mutant F198S, which is also mainly located in the cytoplasm. Monoglycosylated N197D and wild-type PrP, which are mainly attached to the plasma membrane, displayed the weakest PK resistance and aggregation ability (Fig. 4a,c). Our data demonstrate that the fewer glycosylation modifications PrP undergoes, the more likely it is to be located in the cytoplasm and the stronger are its PK resistance and aggregation ability (Fig. 4). Thus, human PrP located in the cytoplasm has much stronger PK resistance and aggregation ability than PrP located on the plasma membrane.

**Figure 5:**
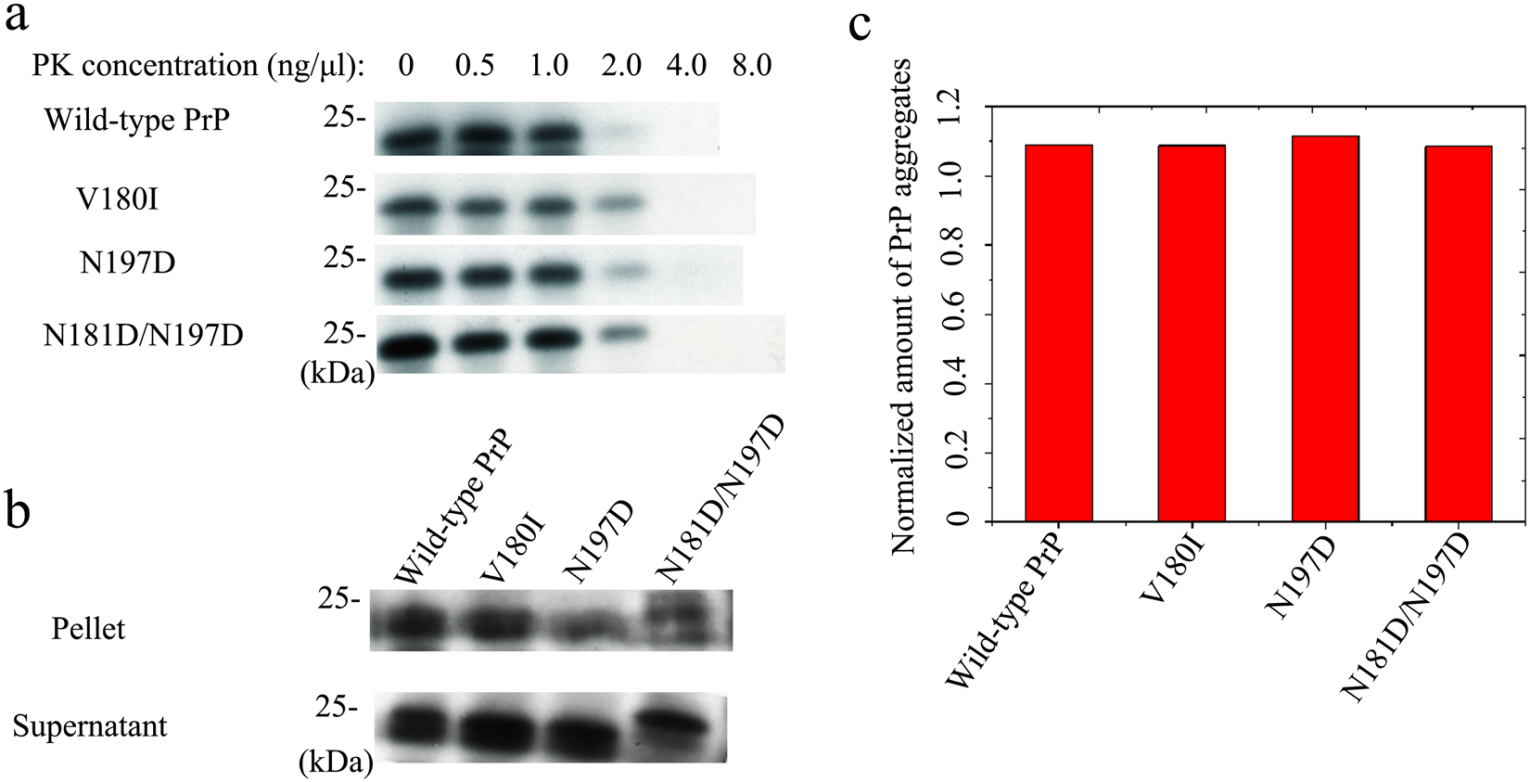
Human PrP displays similar PK resistance and aggregation ability after tunicamycin treatment. RK13 cells stably expressing wild-type PrP, V180I, N197D, or N181D/N197D were cultured for 7 days; tunicamycin was then added to the culture medium 48 h before the cells were harvested. Wild-type PrP and its mutants in RK13 cells treated with tunicamycin were digested with various concentrations of PK (from left to right, 0, 0.5, 1.0, 2.0, 4.0, and 8.0 ng/μl) and probed with the anti-PrP antibody 3F4 **(a)**. The normalized amounts of insoluble PrP aggregates in the stable cells treated with tunicamycin **(c)** were calculated as the ratio of the density of insoluble PrP aggregate bands probed by 3F4 to that of the total PrP bands in cell lysates also probed by 3F4 **(b)**. RK13 cells stably expressing wild-type PrP were used as a control. The gel and blot images of wild-type PrP, V180I, N197D, and N181D/N197D **(a,b)** shown in the figure were cropped from different parts of the same gel with an exposure time of 30 s. Clear delineation with white spaces is used. Full-length blots and gels are presented in Supplementary Figure 6.

RK13 cells stably expressing wild-type or mutant human PrP were cultured for 7 days and treated with tunicamycin for 2 days. The cells were then digested with various concentrations of PK and probed using the anti-PrP antibody 3F4 (Fig. 5a). The normalized amounts of insoluble PrP aggregates in cells treated with tunicamycin (Fig. 5c) were also calculated as the ratio of the density of the immunoreactive band produced by the insoluble PrP aggregate to that of the total PrP band in the cell lysate (25-kDa band, Fig. 5b). Full-length blots and gels are presented in Fig. S5. As can be seen in Figs 5 and S5, wild-type PrP and the V180I, N197D, and N181D/N197D mutants displayed similar PK resistance (25-kDa band, a) and aggregation ability (25-kDa band, b and c) after tunicamycin treatment. Our data demonstrate that N-linked glycosylation is one of the most important factors influencing the PK resistance and aggregation ability of human PrP.

**Figure 6:**
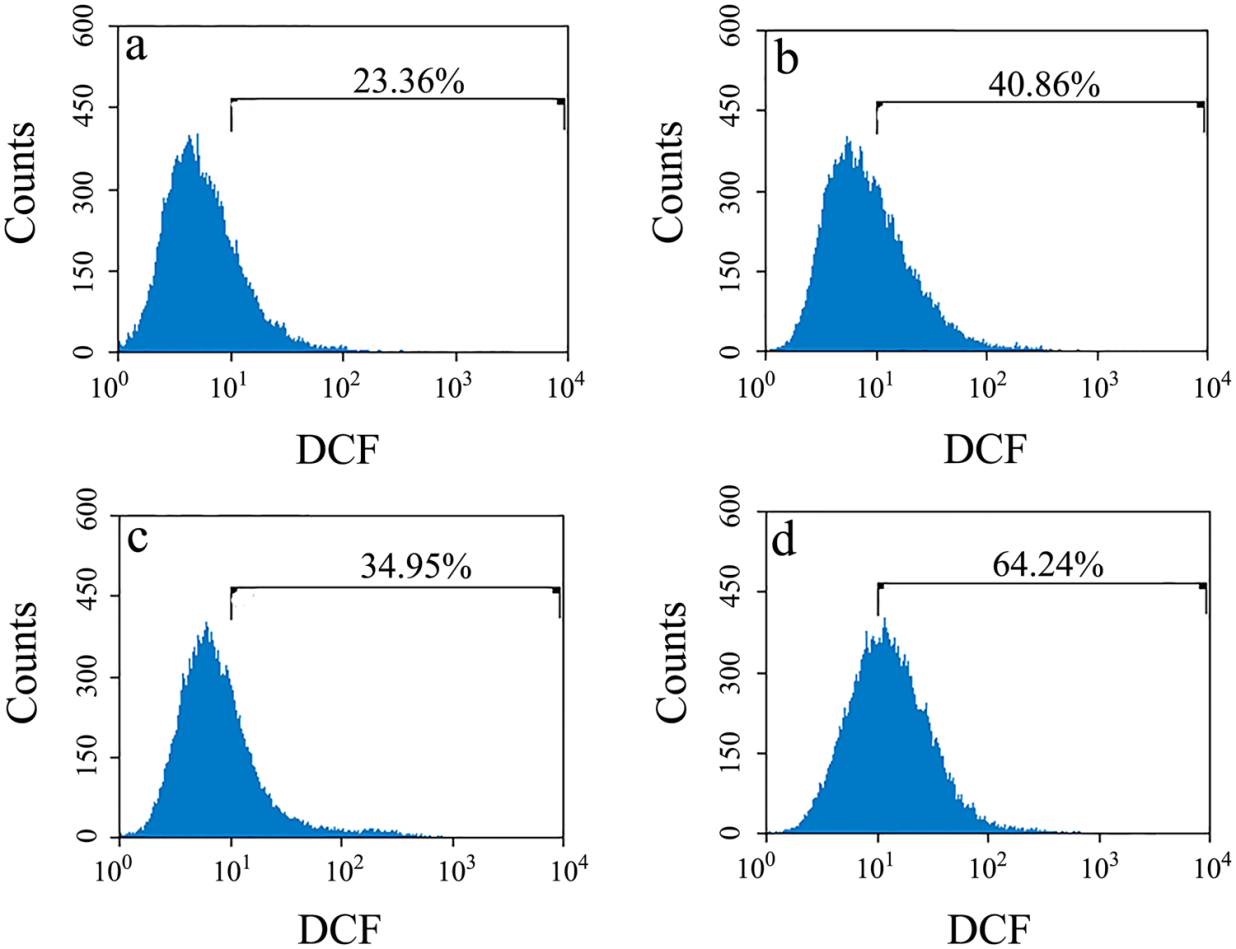
Human PrP located in the cytoplasm of RK13 cells is associated with much higher cellular ROS levels than PrP located on the plasma membrane. RK13 cells stably expressing wild-type PrP **(a)**, V180I **(b)**, N197D **(c)**, or N181D/N197D **(d)** were cultured for 4 days. The percentage of ROS cells was determined by flow cytometry using the ROS probe DCFH-DA. RK13 cells stably expressing wild-type PrP were used as a control.

Next, we used flow cytometry and the ROS probe DCFH-DA to measure ROS levels in RK13 cells stably expressing human PrP and in SH-SY5Y cells transiently expressing human PrP. As shown in Fig. 6, the ROS levels in cells stably expressing wild-type PrP (a), V180I (b), N197D (c), and N181D/N197D (d) were 23.36%, 40.86%, 34.95%, and 64.24%, respectively. As shown in Fig. S6, the ROS levels induced by the toxic prion peptide PrP 106–126 in cells transiently expressing wild-type PrP (a), V180I (b), N197D (c), and N181D/N197D (d) were 8.35%, 20.98%, 11.15%, and 25.29%, respectively. As shown in Fig. S7, SH-SY5Y cells treated with 60 μM PrP 106–126 for 48 h (b) and cells not treated with PrP 106–126 (a) displayed very similar low ROS levels, indicating that the toxic prion peptide PrP 106–126 itself had no obvious effect on the ROS levels in SH-SY5Y cells after 48-h treatment. Clearly, RK13 and SH-SY5Y cells expressing the human PrP mutants N181D/N197D and V180I, which are located in the cytoplasm, contained much higher intracellular ROS levels than RK13 and SH-SY5Y cells expressing wild-type PrP and N197D, which are located on the plasma membrane.

Fig. S8 shows the far-UV CD spectra of 10 μM native wild-type PrP, V180I, N181D, N197D, F198S, and N181D/N197D. Two negative peaks at 208 and 222 nm were observed under all conditions (Fig. S8), indicating that these mutations did not alter the secondary structure of native human PrP. Our CD data demonstrate that the differences in the subcellular location, PK resistance, and aggregation ability of human PrP resulted from its glycosylation rather than from structural changes in the protein.

### N-linked glycosylation deficiency enhances human PrP toxicity in cells induced by MG132 or PrP 106–126

**Figure 7:**
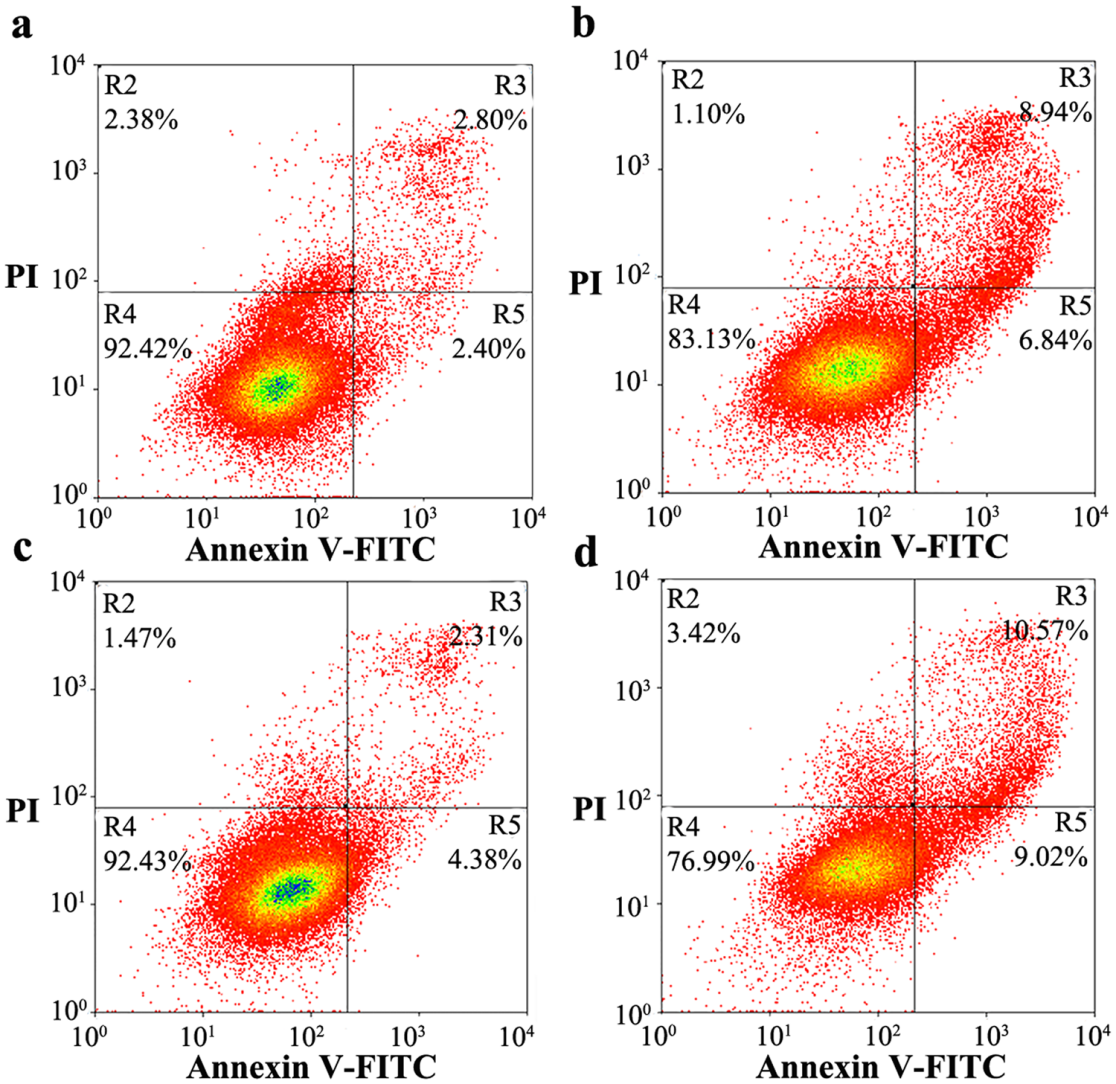
N-linked glycosylation deficiency enhances PrP toxicity induced by MG132 in RK13 cells. RK13 cells stably expressing wild-type PrP **(a)**, V180I **(b)**, N197D **(c)**, or the double mutant N181D/N197D **(d)** were cultured for 3 days and incubated with 1 ng/μl MG132 for 1 day. The percentage of apoptotic cells was determined by flow cytometry. The four quadrants distinguished by annexin V-FITC/PI staining represent viable cells (R4 quadrant), early apoptotic cells (R5 quadrant), late apoptotic cells (R3 quadrant), and operation-damaged cells (R2 quadrant).

**Figure 8:**
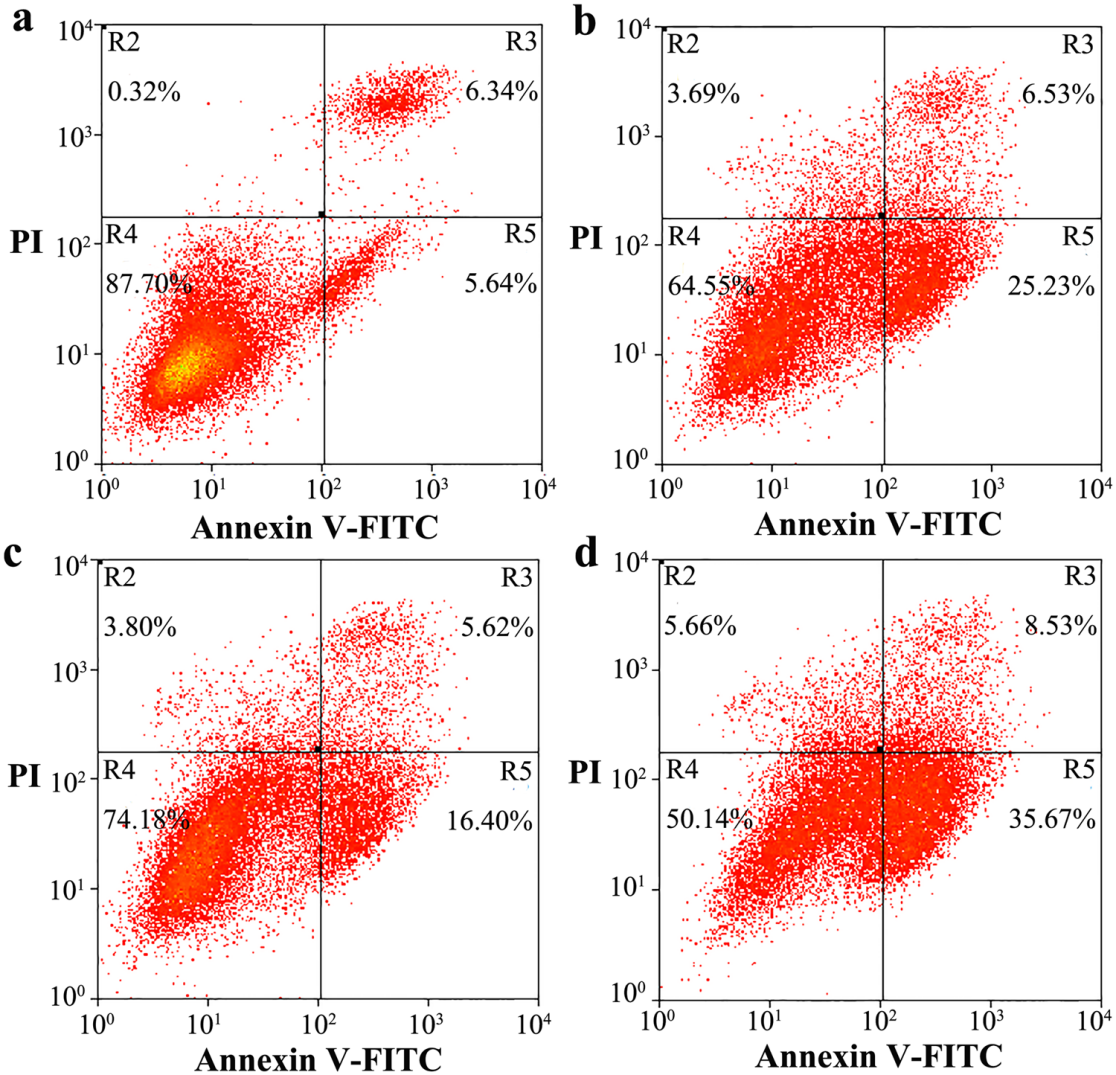
N-linked glycosylation deficiency enhances PrP toxicity in RK13 cells induced by the toxic prion peptide PrP 106–126. RK13 cells stably expressing wild-type PrP **(a)**, V180I **(b)**, N197D **(c)**, or the double mutant N181D/N197D **(d)** were cultured for 3 days and incubated with 60 μM PrP 106–126 for 2 days. The percentage of apoptotic cells was determined by flow cytometry as described in the legend of Fig. 7.

As shown above, glycosylation significantly altered the subcellular localization, PK resistance, and aggregation ability of human PrP. We thus wished to determine whether glycosylation of human PrP directly affects its cytotoxicity. In this paper, we investigated the effects of stably expressed wild-type PrP, N197D, V180I, and N181D/N197D on toxicity in living RK13 cells induced with 1 ng/μl MG132 (Fig. 7) or 60 μM PrP 106–126 (Fig. 8) by flow cytometry with annexin V-FITC and PI staining^9,55–57^. MG132, a proteasome inhibitor^58^, and PrP 106–126, a toxic prion peptide^59,60^, were used to increase the accumulation of PrP aggregates and to induce strong neurotoxicity. As shown in Fig. 7, the percentages of early and late apoptotic cells in living RK13 cells stably expressing V180I treated with 1 ng/μl MG132 for 24 h were 6.84% and 8.94%, respectively (b), and those in living RK13 cells stably expressing N181D/N197D were 9.02% and 10.57%, respectively (d). The percentages of both early and late apoptotic cells were markedly higher than those in RK13 cells incubated with MG132 and stably expressing wild-type PrP (a, 2.40% and 2.80%, respectively). The percentages of early and late apoptotic cells in living RK13 cells treated with MG132 for 24 h and stably expressing N197D were 4.38% and 2.31%, respectively (Fig. 7c), very similar to the percentages in RK13 cells incubated with MG132 and stably expressing wild-type PrP (Fig. 7a). We also used the MTT reduction assay^57^ to measure PrP toxicity in RK13 cells induced by MG132 and stably expressing human PrP. As shown in Fig. S9, after treatment with 1 ng/μl MG132 the viability of RK13 cells stably expressing wild-type PrP (a), V180I (b), N197D (c), and N181D/N197D (d) was 45.8%, 28.2%, 40.4%, and 28.4%, respectively. As shown in Fig. 8, the percentages of early and late apoptotic cells in living RK13 cells treated with 60 μM PrP 106–126 for 2 days and stably expressing V180I were 7.94% and 11.90%, respectively (b), and those in living RK13 cells stably expressing N181D/N197D were 7.88% and 13.47%, respectively (d). In both cases, the percentages of early and late apoptotic cells were markedly greater than the percentages in RK13 cells incubated with PrP 106–126 and stably expressing wild-type PrP (a, 6.18% and 3.97%, respectively). The percentages of early and late apoptotic cells in living RK13 cells treated with PrP 106–126 for 2 days and stably expressing N197D were 8.91% and 7.40%, respectively (Fig. 8c), just slightly greater than those in RK13 cells stably expressing wild-type PrP incubated with PrP 106–126 (Fig. 8a).

**Figure 9:**
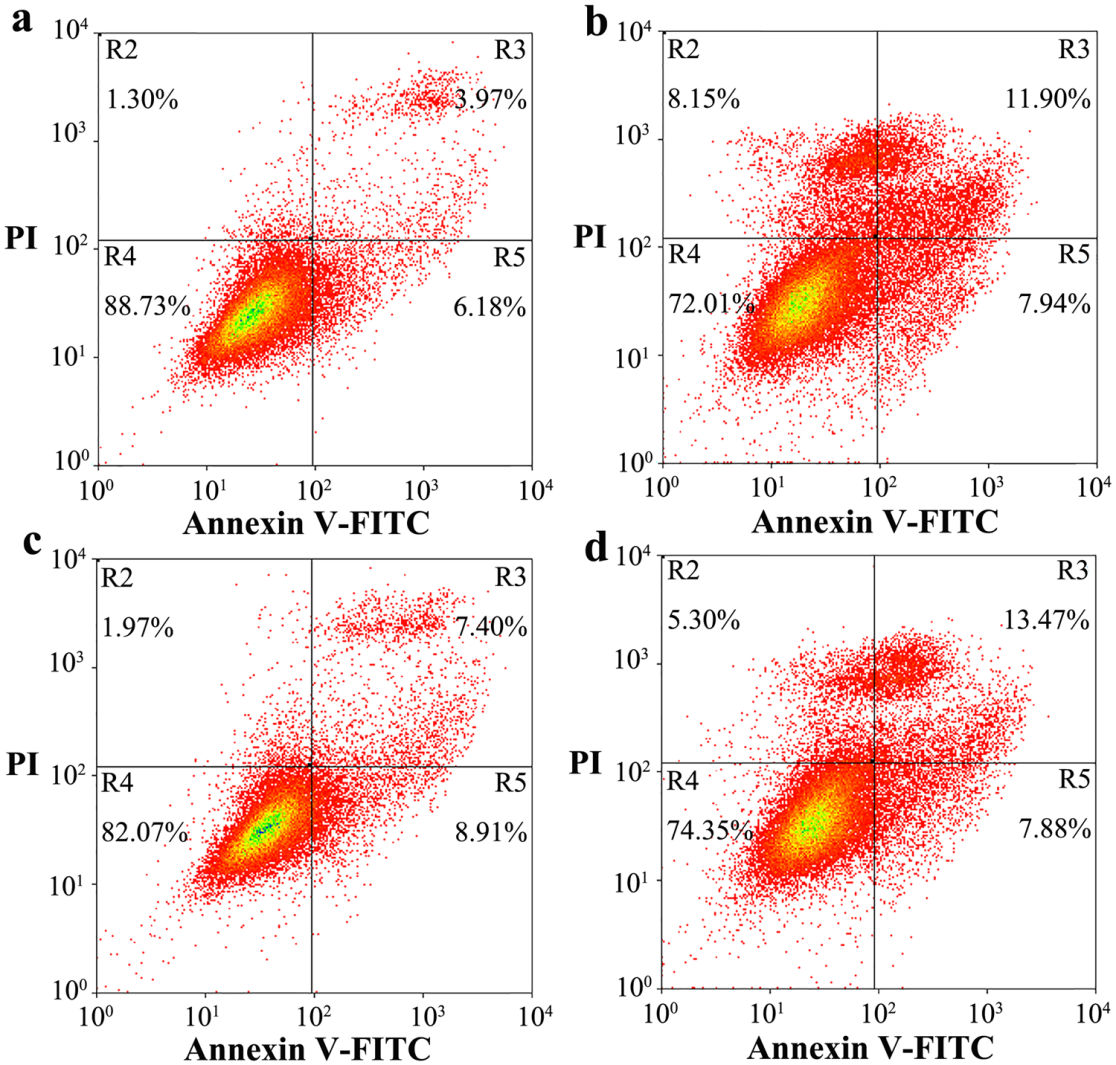
N-linked glycosylation deficiency enhances PrP toxicity in SH-SY5Y cells induced by the toxic prion peptide PrP 106–126. SH-SY5Y cells transiently expressing wild-type PrP **(a)**, V180I **(b)**, N197D **(c)**, or the double mutant N181D/N197D **(d)** were cultured for 2 days and incubated with 60 μM PrP 106–126 for 2 days. The percentage of apoptotic cells was determined by flow cytometry as described in the legend of Fig. 7.

We also investigated the effects of transient expression of wild-type PrP, N197D, V180I, and N181D/N197D on toxicity in living SH-SY5Y cells induced with 60 μM PrP 106–126 by flow cytometry using annexin V-FITC and PI staining (Fig. 9). As shown in Fig. 9, the percentage of early apoptotic cells in living SH-SY5Y cells treated with 60 μM PrP 106–126 for 2 days and transiently expressing V180I was 25.23% (b), and that in living SH-SY5Y cells transiently expressing N181D/N197D was 35.67% (d), both markedly greater than the percentage of early apoptotic cells in SH-SY5Y cells incubated with PrP 106–126 and transiently expressing wild-type PrP (a, 5.64%) or N197D (c, 16.40%). We therefore concluded that unglycosylated N181D/N197D, which is mainly located in the cytoplasm of RK13 and SH-SY5Y cells, has the strongest apoptosis-inducing ability (cytotoxicity), followed by the pathological mutant V180I, which is also mainly located in the cytoplasm. The monoglycosylated mutant N197D, which is mainly attached to the plasma membrane of RK13 and SH-SY5Y cells, and wild-type PrP, most of which is also attached to the plasma membrane, has the weakest cytotoxicity (Figs 7–9 and S9).

The following control experiments were conducted to determine whether MG132 treatment leads directly to apoptosis in RK13 cells. As shown in Fig. S10, RK13 cells treated with 1 ng/μl MG132 for 24 h (b) and those not treated with MG132 (a) displayed similar low levels of apoptosis. As shown in Fig. S11, RK13 cells treated with MG132 at 0.2 ng/μl (b) or 0.4 ng/μl (c) for 72 h, RK13 cells treated with 1 ng/μl MG132 for 24 h and then cultured in minimum essential medium without MG132 for 48 h (d), and RK13 cells not treated with MG132 (a) also displayed similar low levels of apoptosis. The following control experiments were conducted to determine whether PrP 106–126 treatment directly causes apoptosis in RK13 and SH-SY5Y cells. As shown in Fig. S12, cultures of RK13 cells treated with 60 μM PrP 106–126 for 48 h (b) contained a slightly higher percentage of apoptotic cells than cultures that were not treated with PrP 106–126 (a). As shown in Fig. S13, SH-SY5Y cells treated with 60 μM PrP 106–126 for 48 h (b) and SH-SY5Y cells not treated with PrP 106–126 (a) displayed similar low levels of apoptosis. Clearly, treatment with MG132 or PrP 106–126 had no obvious direct effect on apoptosis in RK13 or SH-SY5Y cells on the investigated time scale.

## Discussion

PrP^C^ is a membrane protein that possesses two glycosylation sites and one GPI anchor^19,51^. In this paper, we studied PrP glycosylation and addressed the question of how N-linked glycosylation regulates the subcellular location, aggregation, and toxicity of human PrP for the following reasons. Firstly, although a number of pathological mutants involving the two glycosylation sites of human PrP, such as V180I and F198S^3,5,61–63^, are known to exist, the mechanism behind this phenomenon is largely unknown. Secondly, although glycosylation is one of the most important posttranslational modifications of PrP^42–44^, it is still unclear how glycosylation of PrP affects its functions *in vivo*. It has been reported that unglycosylated PrP provokes apoptosis in cancer cells^21,36^ and that obstruction of glycosylation promotes the acquisition of scrapie-like properties by mouse PrP in Chinese hamster ovary cells^48^. Moreover, the characteristics of TSE strains and the protective mechanisms against PrP misfolding are both determined by glycosylation^64–66^. In this paper, we demonstrated that N-linked glycosylation deficiency not only impairs the correct localization of human PrP at the plasma membrane but also enhances the protein’s PK resistance and aggregation ability, increases cellular ROS levels, and increases the cytotoxicity of human PrP. Thus, we propose that glycosylation functions as a necessary cofactor in determining PrP location on the plasma membrane and that it significantly inhibits the aggregation of human PrP and decreases its cytotoxicity. Our findings could explain why many pathological mutations occur near the glycosylation sites of human PrP.

The main conclusion of the current work is that N-linked glycosylation deficiency impairs the correct localization of human PrP at the plasma membrane. This assertion is supported by our demonstration of the cytoplasmic localization of the N181D/N197D, N181A/N197A, and N181Q/N197Q mutants expressed in RK13 epithelial cells and SH-SY5Y neuroblastoma cells and of wild-type PrP and various PrP mutants expressed in tunicamycin-treated cells. A number of previous studies in which glycosylation-deficient mutants of mouse, hamster, and ovine PrP were expressed in various cell lines or in transgenic mice have produced similar results^45–49^. Here, we report several novel experiments that convincingly show that human PrP requires at least one N-glycan chain to be efficiently expressed at the cell surface, as has previously been shown for murine and ovine PrP^45–49^. This was demonstrated using two different cell types and different amino acid substitutions at the glycosylation sites. This work corrects the erroneous conclusions reached in a recent publication on human PrP in which the N181A/N197A mutant was expressed in Chinese hamster ovary cells^50^. Based on our own data, we suggest that impaired trafficking of mutant human PrP may depend neither on the cell lines employed nor on the specific amino acid substitutions present in the mutant proteins. The other observations of glycosylation-deficient mutants made in this work, including the presence of increased amounts of insoluble aggregates, increased PK resistance and increased cellular levels of ROS, are also convincing. In future studies, we will perform cell biological experiments to determine whether unglycosylated cytoplasmic PrP is cytotoxic per se, i.e., whether it produces simultaneous metabolic impairment of the cells.

**Figure 10:**
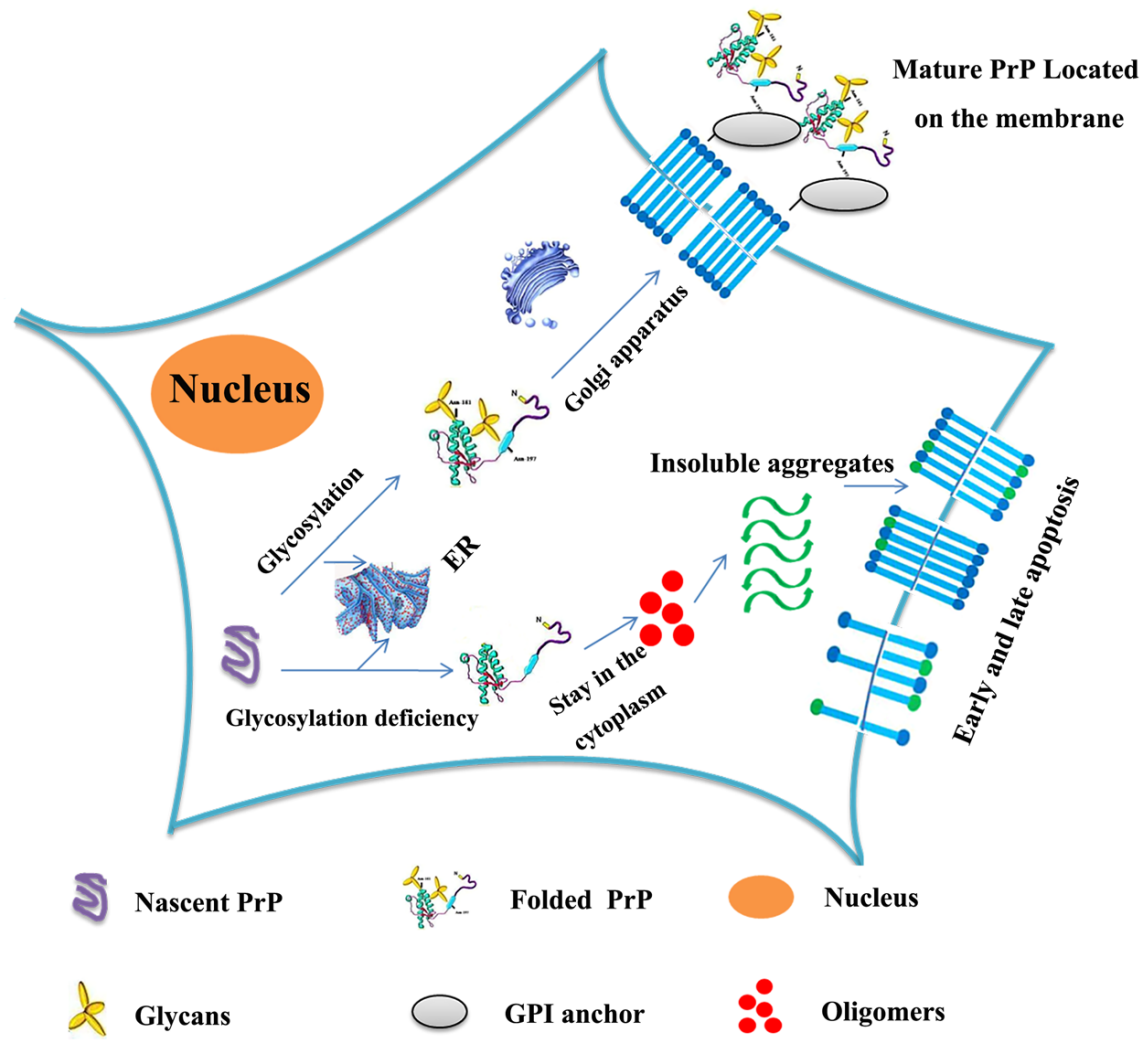
A hypothetical model showing how glycosylation inhibits the aggregation of human PrP and decreases PrP toxicity in living cells. Under normal circumstances, posttranslational modification of nascent PrP (purple unfolded PrP), including N-linked glycosylation, occurs in the ER; the modified, folded PrP (green folded PrP modified by yellow glycans) then matures in the Golgi apparatus and finally reaches the plasma membrane with the help of the GPI anchor (gray ellipse). However, N-linked glycosylation deficiency causes PrP to remain in the cytoplasm and to have stronger PK resistance and aggregation ability than mature PrP located on the plasma membrane. Glycosylation deficiency also promotes early and late apoptosis induced by PrP oligomers (red balls) and insoluble PrP aggregates (green waves) or by the metabolic consequences of this aggregation, thereby increasing PrP toxicity. Externalized phosphatidylserine is indicated by blue sticks with green balls.

Figure 10 presents a hypothetical model of how glycosylation might inhibit the aggregation of human PrP and decrease PrP toxicity in living cells. Under normal circumstances, posttranslational modifications of nascent PrP, such as N-linked glycosylation, occur in the ER; the modified, folded PrP subsequently matures in the Golgi apparatus and finally reaches the plasma membrane with the help of the GPI anchor. However, N-linked glycosylation deficiency causes human PrP to remain in the cytoplasm and prevents the correct localization of human PrP at the plasma membrane. Furthermore, human PrP located in the cytoplasm has much stronger PK resistance and aggregation ability and produces much higher cellular ROS levels than PrP located on the plasma membrane, and glycosylation deficiency promotes early and late apoptosis and enhances the cytotoxicity of human PrP. These findings add to our current knowledge by showing that glycosylation deficiency impairs the correct localization of PrP at the plasma membrane and enhances the tendency of the normally folded cellular form of the protein, PrP^C^, to transform into the pathologic form, PrP^Sc^. Our findings help explain how posttranslational modification regulates the formation of disease-causing structures by human PrP *in vivo* and should be useful in the development of new therapeutic reagents for prion diseases.

## Methods

### Reagents

Three dyes, ThT, 3-(4,5-dimethylthiazol-2-yl)-2,5-diphenyltetrazolium bromide (MTT) and DAPI, and two antibodies, the mouse anti-PrP monoclonal antibody 3F4^41,48^ and a 10-nm gold-labeled anti-mouse antibody^7,9,57^, were purchased from Sigma-Aldrich (St. Louis, MO). Sarkosyl and PK were obtained from Amresco (Solon, OH). Alexa 546-conjugated fluorescent secondary antibody^7,9,57^, the proteasome inhibitor MG132, and the ROS probe DCFH-DA (2′,7′-dichlorofluorescein diacetate) were purchased from Beyotime (Nantong, China), respectively. Ni-Sepharose was obtained from GE Company (Pittsburgh, PA). All other chemicals used in this study were of analytical grade and were produced in China.

### PrP expression and purification

Wild-type human PrP (23–231) and its mutants V180I, F198S, N181D, N197D, and N181D/N197D were inserted into the pET30a plasmid. N181D/N197D was generated by polymerase chain reaction (PCR) using the wild-type PrP plasmid as a template with two pairs of primers (5′CTTGATTGTGATATCGACGCAGTCGTGC/5′GCACGACTGCGTCGATATCACAATCAAG (N181D) and 5′GGTCTCGGTGAAGTCCTCCCCCTTGGTG /5′CACCAAGGGGGAGGACTTCACCGAGACC (N197D)). The template DNA was digested by the DpnI restriction enzyme at 37 °C for 3 h. The plasmids for wild-type PrP and its mutants were transformed into *Escherichia coli*. Recombinant wild-type human PrP and its mutant were expressed in *E*. *coli* BL21 (DE3) (Novagen, Merck, Darmstadt, Germany) and purified by HPLC on a C4 reverse-phase column (Shimadzu, Kyoto, Japan) as described by Baskakov^67^ and Liang^10,68^. After purification, human PrP was dialyzed against 10 mM HEPES buffer containing 20 mM NaAc (pH 7.0) for 24 h, concentrated, filtered, and stored at −80 °C. Purified human PrP was shown by SDS-PAGE and mass spectrometry to be a single species with an intact disulfide bond. The concentration of human PrP was determined from its absorbance at 280 nm using the molar extinction coefficient value calculated from the composition of the protein (http://web.expasy.org/protparam/).

### Cell culture

The RK13 and SH-SY5Y cell lines were cultured in 5% CO_2_ at 37 °C in minimum essential medium (Gibco, Invitrogen, Mulgrave, VIC, Australia) and in Dulbecco’s modified Eagle’s medium, respectively, supplemented with 10% (v/v) fetal bovine serum (Gibco), 100 U/ml penicillin, and 100 U/ml streptomycin.

RK13 cell lines stably expressing wild-type human PrP or its mutants V180I, N197D, F198S, and N181D/N197D (“stable cells”) were constructed using a lentiviral vector construction system. The target DNA fragments were cloned into the lentiviral vector pHAGE-puro at the BamH I and Xhol I restriction sites. The lentiviral vector construction system containing the CMV promoter was packaged in HEK293T cells using various combinations of plasmids and liposomes. The three plasmids pHAGE-wild-type PrP (or pHAGE-V180I, pHAGE-N197D, pHAGE-F198S, or pHAGE-N181D/N197D), pVSVG, and pLP were mixed at a ratio of 2:1:1; the ratio of liposomes to DNA was 2:1. After 48 h of transfection, the viruses were harvested and filtered. RK13 cells were infected by the packaged lentivirus with a high infection efficiency, and the expression of individual proteins was detected by Western blotting.

### Circular dichroism spectroscopy

CD spectra were obtained on a Jasco J-810 spectropolarimeter (Jasco Corp., Tokyo, Japan) with a thermostatted cell holder. A quartz cell with a 1-mm light path was used for measurements in the far-UV region. Spectra of 10 μM native wild-type PrP and its mutants V180I, N181D, N197D, F198S, and N181D/N197D in 10 mM HEPES buffer containing 20 mM NaAc (pH 7.0) were recorded from 195 to 250 nm for far-UV CD. Measurements were made at 25 °C. The spectra of all scans were corrected relative to the buffer blank. The mean residue molar ellipticity [θ] (deg · cm^2^ · dmol^−1^) was calculated using the formula [θ] = (*θ*_obs_/10)(MRW/*lc*), where *θ*_obs_ is the observed ellipticity in deg, MRW is the mean residue molecular weight (109.2 Da for human PrP), *l* is the path length in cm, and *c* is the protein concentration in g/ml.

### Laser scanning confocal analysis

RK13 cells and SH-SY5Y cells were transiently transfected with wild-type human PrP or its mutants V180I, N181D, N181A, N197D, N197A, F198S, N181D/N197D, N181A/N197A, N181Q/N197Q, and T199N/N181D/N197D in the pcDNA 3.1 vector using Lipofectamine® 2000 (Invitrogen, Carlsbad, CA) according to the manufacturer’s instructions. The cells were harvested 36 h after transfection and grown on glass coverslips in germ-free 12-well cell culture plates. After 3 days or after 3 days followed by 36 h of treatment with 5 μg/ml tunicamycin, the transient cells and the stable cells were fixed with 4% paraformaldehyde for 30 min, permeabilized with 0.25% Triton-X 100 for 5 min, and immunostained with the primary monoclonal antibody 3F4 for 2 h at 37 °C and with an Alexa Fluor 546-linked secondary antibody for 45 min at 37 °C. The nuclei were stained with DAPI for 5 min at 37 °C, and the cells were visualized by confocal microscopy to permit observation of the subcellular localization of human PrP. Images were captured using an Olympus FluoView FV1000 laser scanning confocal microscope (Tokyo, Japan). Blue fluorescence was detected using the 405-nm laser line of a 405–30 diode laser, and red fluorescence was detected using the 559-nm laser line of the HeNe laser.

### Western blotting

RK13 cell lines stably expressing wild-type human PrP or its mutants V180I, N197D, F198S, and N181D/N197D were cultured in minimum essential medium (Gibco). After 7 days or after 7 days followed by 2 days of treatment with 5 μg/ml tunicamycin, the cells were harvested, lysed in lysis buffer containing 1% Triton X-100, 50 mM Tris, 150 mM NaCl, 1 mM phenylmethanesulfonyl fluoride, and protease inhibitors (pH 7.6) on ice for 30 min, broken by ultrasonication for 1 min, and centrifuged at 10,000 *g* for 10 min. The resultant supernatant fractions were incubated with 1% sarkosyl for 30 min at room temperature with agitation; the detergent-insoluble proteins were then collected by ultracentrifugation of the supernatants at 150,000 *g* for 30 min and washed twice with PBS buffer. For Western blot assays, whole cell lysates and ultracentrifugation pellets were boiled in SDS-PAGE loading buffer, subjected to 13.5% SDS-PAGE and transferred to polyvinylidene difluoride membranes (Millipore, Billerica, MA). The membranes were blocked with 5% fat-free milk in 100 mM Tris-HCl buffer containing 150 mM NaCl, 3 mM KCl, and 0.05% Tween 20 and incubated with the mouse monoclonal anti-PrP antibody 3F4 (1/10,000) for 1 h at room temperature followed by incubation with a homologous horseradish peroxidase-conjugated secondary antibody at a dilution of 1:5000 for 1 h at room temperature. The protein amounts of the loading samples were normalized using the BCA Protein Quantification Kit (Beyotime). Immunoreactive bands were visualized using enhanced chemiluminescence (BeyoECL Plus, Beyotime). The normalized amounts of insoluble PrP aggregates in the stable cells were calculated as the ratio of the density of insoluble PrP aggregate bands probed with the 3F4 antibody to that of the total PrP bands in cell lysates also probed with 3F4. RK13 cells stably expressing wild-type PrP were used as a control for Western blotting. All Western blotting experiments were repeated three times. Statistical analyses were performed using Student’s *t*-test. Values of *p* < 0.05 were considered to indicate statistical significance. The following notation is used throughout: *p < 0.05, **p < 0.01, ***p < 0.001 relative to the control.

### Image acquisition tools and image processing software packages

The digital images were obtained using an EPSON Perfection V750 PRO scanner (Seiko Epson Corporation, Suwa, Japan). Densitometry was performed using ImageJ software (NIH, Bethesda, MD).

### PK digestion assays

RK13 cell lines stably expressing wild-type human PrP or its mutants V180I, N197D, F198S, and N181D/N197D were cultured in minimum essential medium (Gibco). After 7 days or after 7 days followed by 2 days of treatment with 5 μg/ml tunicamycin, the stable cells were harvested and lysed in mild lysis buffer containing 50 mM Tris, 0.5% sodium deoxycholate, and 0.5% Triton X-100 (pH 7.4). Wild-type PrP and its mutants in RK13 cells were digested by 0–8.0 ng/μl PK for 30 min at 37 °C. Digestion was terminated by the addition of 2 mM phenylmethylsulfonyl fluoride (PMSF) and incubation in boiling water for 15 min. The cell lysate was boiled in SDS-PAGE loading buffer, subjected to 13.5% SDS-PAGE and transferred to polyvinylidene difluoride membranes (Millipore). The membranes were blocked with 5% fat-free milk in 100 mM Tris-HCl buffer containing 150 mM NaCl, 3 mM KCl, and 0.05% Tween 20. The membranes were incubated with the mouse monoclonal anti-PrP antibody 3F4 (1/10,000) for 1 h at room temperature followed by incubation with a homologous horseradish peroxidase-conjugated secondary antibody at a dilution of 1:5000 for 1 h at room temperature.

### Annexin V-FITC apoptosis detection assay

Apoptotic cells were detected by flow cytometry after staining with an annexin V-FITC apoptosis detection kit (Beyotime). Briefly, RK13 cells or RK13 cells stably expressing wild-type PrP, V180I, N197D, or the double mutant N181D/N197D were cultured in minimum essential medium for 3 days and incubated with 1 ng/μl MG132 for 1 day or 60 μM PrP 106–126 for 2 days; SH-SY5Y cells or SH-SY5Y cells transiently expressing wild-type PrP, V180I, N197D, or double mutant N181D/N197D were cultured in Dulbecco’s modified Eagle’s medium for 2 days, incubated with 60 μM PrP 106–126 for 2 days, digested in 2.5 mg/ml trypsin (Promega, Madison, WI) and harvested. RK13 cells cultured in minimum essential medium for 3 days, incubated with 0.2 or 0.4 ng/μl MG132 for 3 days or with 1 ng/μl MG132 for 1 day and then cultured in minimum essential medium without MG132 for 2 days or with 60 μM PrP 106–126 for 2 days were also digested in 2.5 mg/ml trypsin and harvested. The cells were collected by centrifugation at 1,000 g for 5 min, washed with PBS buffer and resuspended in 185 μl of binding buffer; the samples were then incubated with 5 μl annexin V-FITC and 10 μl propidium iodide (PI) for 10 min at 4 °C in the dark. Annexin V binding was analyzed using an EPICS XL-MCL flow cytometer (Beckman Coulter, Fullerton, CA). The percentage of apoptotic cells was calculated from the total (~3 × 10^4^ cells) using EXPO32 MultiComp software (Beckman Coulter). Viable cells did not bind annexin V or PI (lower left quadrant R4), early apoptotic cells bound annexin V but not PI (lower right quadrant R5), and late apoptotic cells were both annexin V- and PI-positive (upper right quadrant R3). The upper left quadrant R2 contains cells that were damaged during preparation of the cell suspension. All apoptotic blot experiments were repeated three times.

### Cell viability assays

RK13 cell lines stably expressing wild-type PrP, V180I, N197D, or the double mutant N181D/N197D were plated in 96-well plates in minimum essential medium for 3 days and incubated with MG132 for 1 day for cell viability assays. After incubation for 4 days, the cells were washed with PBS buffer, and fresh medium containing 10% (v/v) fetal bovine serum and MTT was added to yield a final concentration of 0.5 mg/ml (MTT); the cells were then incubated for an additional 4 h. The medium was then replaced with 150 μl of pure dimethyl sulfoxide, and the absorbance of the dark blue formazan at 492 nm was measured using a Thermo Multiskan MK3 microplate reader (Thermo Scientific, Waltham, MA). Cell viability was expressed as the percentage ratio of the absorbance of wells containing the treated samples to that of wells containing cells not treated with MG132. The data on cell viability are expressed as the mean ± S.D. of the values obtained in 5 independent experiments. Statistical analyses were performed using Student’s *t*-test. Values of *p* < 0.05 were considered to indicate statistical significance. The following notation is used throughout: *p < 0.05, **p < 0.01, ***p < 0.001 relative to the control.

### Oxidative stress detection

RK13 cells stably expressing wild-type PrP, V180I, N197D, or the double mutant N181D/N197D were cultured in minimum essential medium for 4 days. SH-SY5Y cells transiently expressing wild-type PrP, V180I, N197D, or the double mutant N181D/N197D were cultured in Dulbecco’s modified Eagle’s medium for 2 days and incubated with 60 μM PrP 106–126 for 2 days. An ROS probe, DCFH-DA, at a starting concentration of 10 mM was diluted to 1:1200 in minimum essential medium or Dulbecco’s modified Eagle’s medium and added to the stable cells or the transient cells, and the treated cells were placed in a CO_2_ incubator for 20 min. After the incubation, the cells were washed twice with PBS buffer, digested in 2.5 mg/ml trypsin (Promega) and collected by centrifugation at 1,000 g for 5 min. The cells were then resuspended in PBS buffer, filtered through carbasus, and placed in darkness for 10 min at 4 °C. The percentage of the total number of cells (~15,000) containing ROS was determined on an EPICS XL-MCL flow cytometer (Beckman Coulter) using EXPO32 MultiComp software (Beckman Coulter). RK13 cells stably expressing wild-type PrP were used as a control.

## Electronic supplementary material

Supplemental Data

## Data Availability

All of the data referenced in this manuscript are available.
