# Author Correction: Glycosylation Significantly Inhibits the Aggregation of Human Prion Protein and Decreases Its Cytotoxicity

**DOI:** 10.1038/s41598-018-31650-9

**Published:** 2018-09-04

**Authors:** Chuan-Wei Yi, Li-Qiang Wang, Jun-Jie Huang, Kai Pan, Jie Chen, Yi Liang

**Affiliations:** State Key Laboratory of Virology, College of Life Sciences, Wuhan University, Wuhan, 430072 China

Correction to: *Scientific Reports* 10.1038/s41598-018-30770-6, published online 22 August 2018

This Article contains an error in the order of the Figures. Figures 8 and 9 were published as Figures 9 and 8 respectively. The correct Figures 8 and 9 appear below as Figures 1 and 2. The Figure legends are correct.

**Figure 1:**
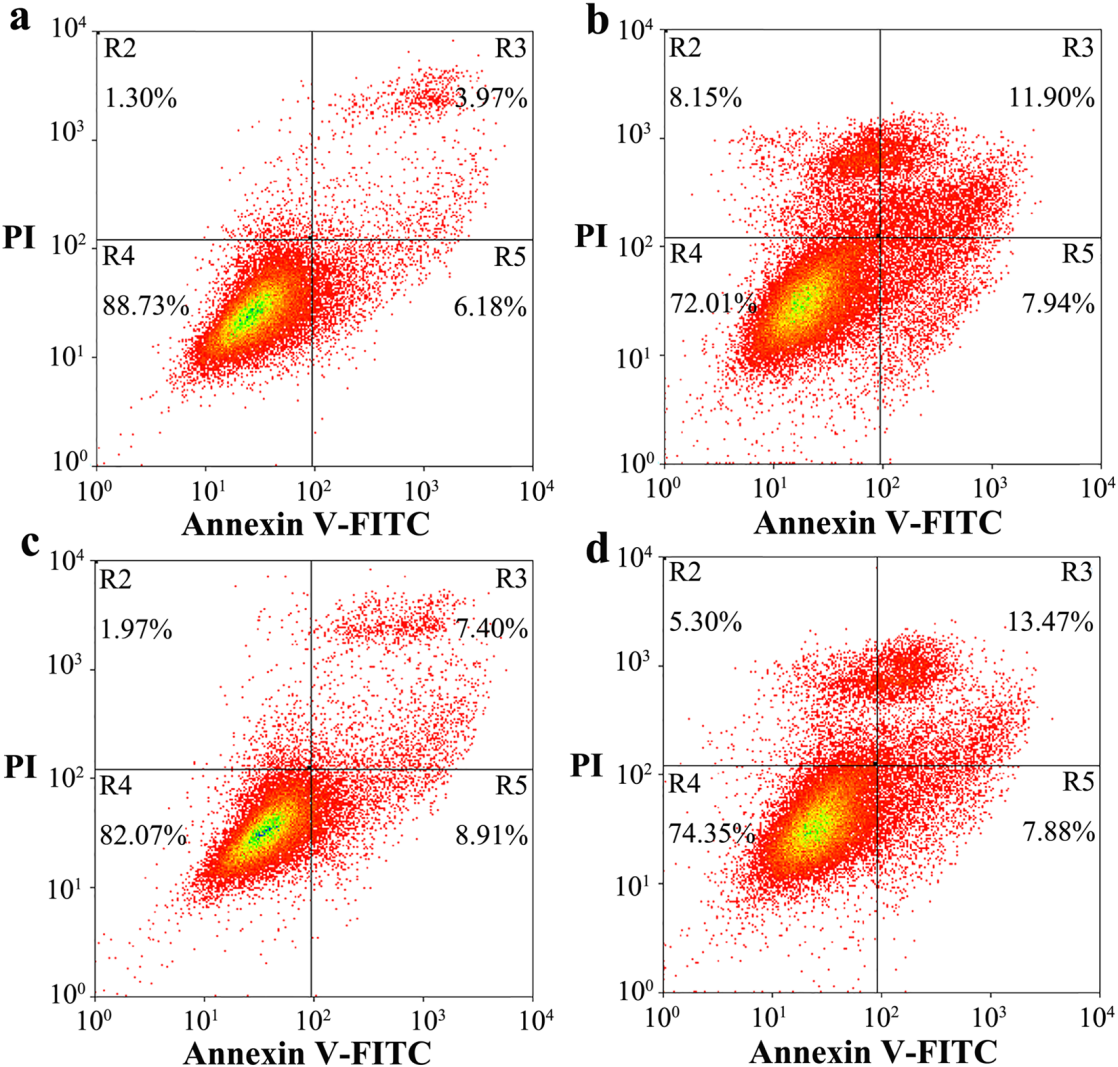
N-linked glycosylation deficiency enhances PrP toxicity in RK13 cells induced by the toxic prion peptide PrP 106–126. RK13 cells stably expressing wild-type PrP (**a**), V180I (**b**), N197D (**c**), or the double mutant N181D/N197D (**d**) were cultured for 3 days and incubated with 60 μM PrP 106–126 for 2 days. The percentage of apoptotic cells was determined by flow cytometry as described in the legend of Fig. 7.

**Figure 2:**
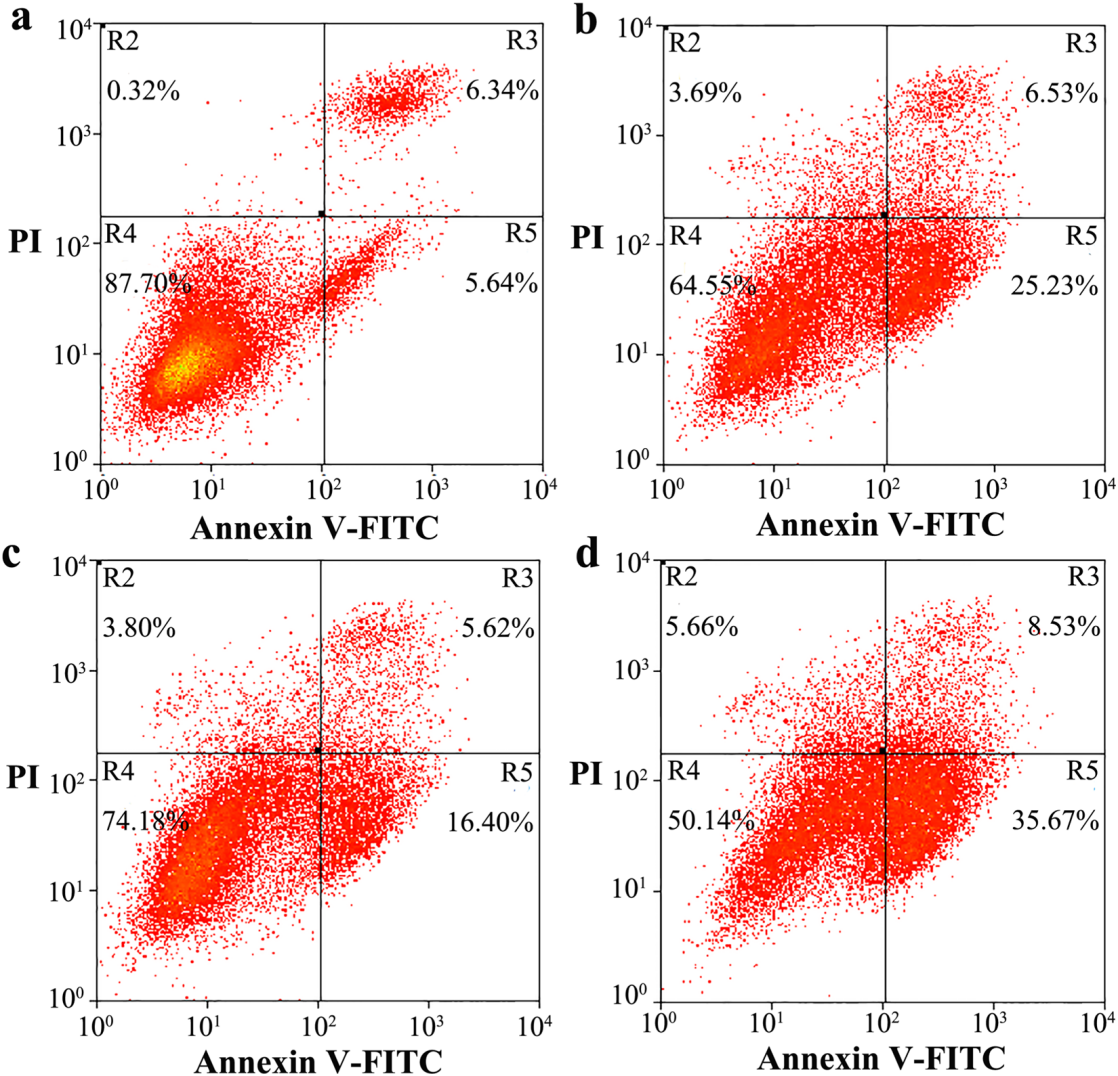
N-linked glycosylation deficiency enhances PrP toxicity in SH-SY5Y cells induced by the toxic prion peptide PrP 106–126. SH-SY5Y cells transiently expressing wild-type PrP (**a**), V180I (**b**), N197D (**c**), or the double mutant N181D/N197D (**d**) were cultured for 2 days and incubated with 60 μM PrP 106–126 for 2 days. The percentage of apoptotic cells was determined by flow cytometry as described in the legend of Fig. 7.

