# Supplementary material for: Glycosylation Significantly Inhibits the Aggregation of Human Prion Protein and Decreases Its Cytotoxicity
Source: Sci Rep. 2018 Aug 22;8:12603. doi: 10.1038/s41598-018-30770-6 (PMC6105643; doi:10.1038/s41598-018-30770-6)
Supplement: Supplementary file 1 — Supplemental Data [file 41598_2018_30770_MOESM1_ESM.pdf]

Glycosylation Significantly Inhibits the Aggregation of Human Prion Protein and Decreases Its Cytotoxicity

Chuan-Wei Yi, Li-Qiang Wang, Jun-Jie Huang, Kai Pan, Jie Chen & Yi Liang *

State Key Laboratory of Virology, College of Life Sciences, Wuhan University, Wuhan 430072, China

* Correspondence and requests for materials should be addressed to Y.L. (liangyi@whu.edu.cn)

Supplementary information

Figure S1. N-linked glycosylation deficiency impairs the correct localization of human PrP at the plasma membrane of RK13 cells. RK13 cells stably expressing wild-type PrP (a-d), V180I (e-h), F198S (i-l), N181D (m-p), N197D (q-t), and N181D/N197D (u-x) were cultured for 2 days at 37°C, fixed, permeabilized, immunostained with the anti-PrP antibody 3F4 with IgG conjugated to Alexa Fluor 546 (red), stained with DAPI (blue), and observed by confocal microscopy. The scale bars represent 10 μm.

Figure S2. N-linked glycosylation deficiency also impairs the correct localization of human PrP at the plasma membrane of SH-SY5Y cells. SH-SY5Y cells transiently expressing wild-type PrP (a-d), V180I (e-h), F198S (i-l), N181D (m-p), N197D (q-t), and N181D/N197D (u-x) were cultured for 2 days at 37°C, fixed, permeabilized, immunostained with the anti-PrP antibody 3F4 with IgG conjugated to Alexa Fluor 546 (red), stained with DAPI (blue), and observed by confocal microscopy. The scale bars represent 10 μm.

Figure S3. N-linked glycosylation deficiency impairs the correct localization of human PrP at the plasma membrane of SH-SY5Y cells. SH-SY5Y cells transiently expressing wild-type PrP (a-d), N181A (e-h), N197A (i-l), N181A/N197A (m-p), and N181Q/N197Q (q-t) were cultured for 2 days at 37°C, fixed, permeabilized, immunostained with the anti-PrP antibody 3F4 with IgG conjugated to Alexa Fluor 546 (red), stained with DAPI (blue), and observed by confocal microscopy. The scale bars represent 10 μm.

Figure S4. Full-length blots and gels of human PrP in the stable cells. RK13 cells stably expressing wild-type PrP, V180I, N197D, F198S, or N181D/N197D were cultured for 7 days. Wild-type PrP and its mutants in RK13 cells were digested with various concentrations of PK (from right to left, 0, 0.5, 1.0, 2.0, 4.0, and 8.0 ng/μl) and probed with the anti-PrP antibody 3F4 (a). Insoluble PrP aggregate bands in the pellet of the stable cells and the total PrP bands in cell lysates, probed by 3F4 (b). The diglycosylated, monoglycosylated, and unglycosylated bands are referred to as “di-”, “mono-”, and “un-”, respectively, and are annotated in (b). The gels and blots of wild-type PrP and V180I (a) were cropped from different parts of the same gel. Similarly, the gels and blots of N197D, F198S, or N181D/N197D (a) were cropped from different parts of the same gel. The images of wild-type PrP (or V180I) and N197D (or F198S or N181D/N197D) shown were obtained from two different gels using the same exposure time (30 s) (a). The gels and blots of wild-type PrP, V180I, N197D, F198S, or N181D/N197D (b) were cropped from different parts of the same gel with an exposure time of 30 s. Clear delineation with white spaces was used.

Figure S5. Full-length blots and gels of human PrP in the stable cells when treated with tunicamycin. RK13 cells stably expressing wild-type PrP, V180I, N197D, or N181D/N197D were cultured for 7 days; tunicamycin was then added to the culture medium 48 h before the cells were harvested. Wild-type PrP and its mutants in RK13 cells treated with tunicamycin were digested with various concentrations of PK (from left to right, 0, 0.5, 1.0, 2.0, 4.0, and 8.0 ng/μl) and probed with the anti-PrP antibody 3F4 (a). Insoluble PrP aggregate bands in the pellet of the stable cells probed by 3F4 and the total PrP bands in cell lysates also probed by 3F4 (b). RK13 cells stably

expressing wild-type PrP were used as a control. Whole gel of wild-type PrP, V180I, N197D, or N181D/N197D with an exposure time of 30 s is shown in (a). The gels and blots of wild-type PrP, V180I, N197D, or N181D/N197D (b) were cropped from different parts of the same gel with an exposure time of 30 s. Clear delineation with white spaces was used.

Figure S6. Human PrP located in the cytoplasm of SH-SY5Y cells induced by the toxic prion peptide PrP 106-126 is associated with much higher cellular ROS levels than PrP located on the plasma membrane. SH-SY5Y cells transiently expressing wild-type PrP (a), V180I (b), N197D (c), or the double mutant N181D/N197D (d) were cultured for 2 days and incubated with 60 μM PrP 106-126 for 2 days. The percentage of ROS cells was determined by flow cytometry using the ROS probe DCFH-DA. SH-SY5Y cells transiently expressing wild-type PrP were used as a control.

Figure S7. The toxic prion peptide PrP 106-126 itself has no obvious effect on the ROS levels in SH-SY5Y cells after 48-h treatment. SH-SY5Y cells treated with 60 μM PrP 106-126 for 48 h (b) and cells not treated with PrP 106-126 (a) displayed very similar low ROS levels. The percentage of ROS cells was determined by flow cytometry as described in the legend of Fig. S6.

Figure S8. Secondary structure of native human PrP. The far-UV CD spectra of 10 μM native wild-type PrP (black), V180I (red), N181D (green), N197D (blue), F198S (cyan), and N181D/N197D (magenta). Two negative peaks at 208 and 222 nm were observed under all conditions, indicating that these mutations did not alter the

secondary structure of native human PrP.

Figure S9. PrP toxicity induced by MG132 in RK13 cells. RK13 cells stably expressing wild-type PrP (a), V180I (b), N197D (c), or the double mutant N181D/N197D (d) were cultured for 3 days and incubated with 1 ng/μl MG132 for 1 day. The cytotoxicity was measured by the MTT reduction assay. Cells not treated with MG132 were used as a control. The data on cell viability are expressed as the mean ± S.D. of the values obtained in 5 independent experiments. Statistical analyses were performed using Student's *t*-test. Values of $p < 0.05$ were considered to indicate statistical significance. The following notation is used throughout: ∗, $p < 0.05$, ∗∗, $p < 0.01$, ∗∗∗, $p < 0.001$ relative to the control.

Figure S10. MG132 itself has no obvious effect on apoptosis in RK13 cells after 24-h treatment. RK13 cells treated with 1 ng/μl MG132 for 24 h (b) and those not treated with MG132 (a) displayed similar low levels of apoptosis. The percentage of apoptotic cells was determined by flow cytometry. The four quadrants distinguished by annexin V-FITC/PI staining represent viable cells (R4 quadrant), early apoptotic cells (R5 quadrant), late apoptotic cells (R3 quadrant), and operation damaged cells (R2 quadrant).

Figure S11. MG132 itself has no obvious effect on apoptosis in RK13 cells after 72-h treatment. RK13 cells treated with 0.2 ng/μl (b) and 0.4 ng/μl (c) MG132 for 72 h, RK13 cells treated with 1 ng/μl MG132 for 24 h and then cultured in minimum essential medium without MG132 for 48 h (d), and RK13 cells not treated with MG132 (a) displayed similar low levels of apoptosis. The percentage of apoptotic

cells was determined by flow cytometry as described in the legend of Fig. S10.

Figure S12. The toxic prion peptide PrP 106-126 itself has no obvious effect on apoptosis in RK13 cells after 48-h treatment. Cultures of RK13 cells treated with 60 μM PrP 106-126 for 48 h (b) contained a slightly higher percentage of apoptotic cells than cultures that were not treated with PrP 106-126 (a). The percentage of apoptotic cells was determined by flow cytometry as described in the legend of Fig. S10.

Figure S13. The toxic prion peptide PrP 106-126 itself has no obvious effect on apoptosis in SH-SY5Y cells after 48-h treatment. SH-SY5Y cells treated with 60 μM PrP 106-126 for 48 h (b) and SH-SY5Y cells not treated with PrP 106-126 (a) displayed similar low levels of apoptosis. The percentage of apoptotic cells was determined by flow cytometry as described in the legend of Fig. S10.

Figure S1

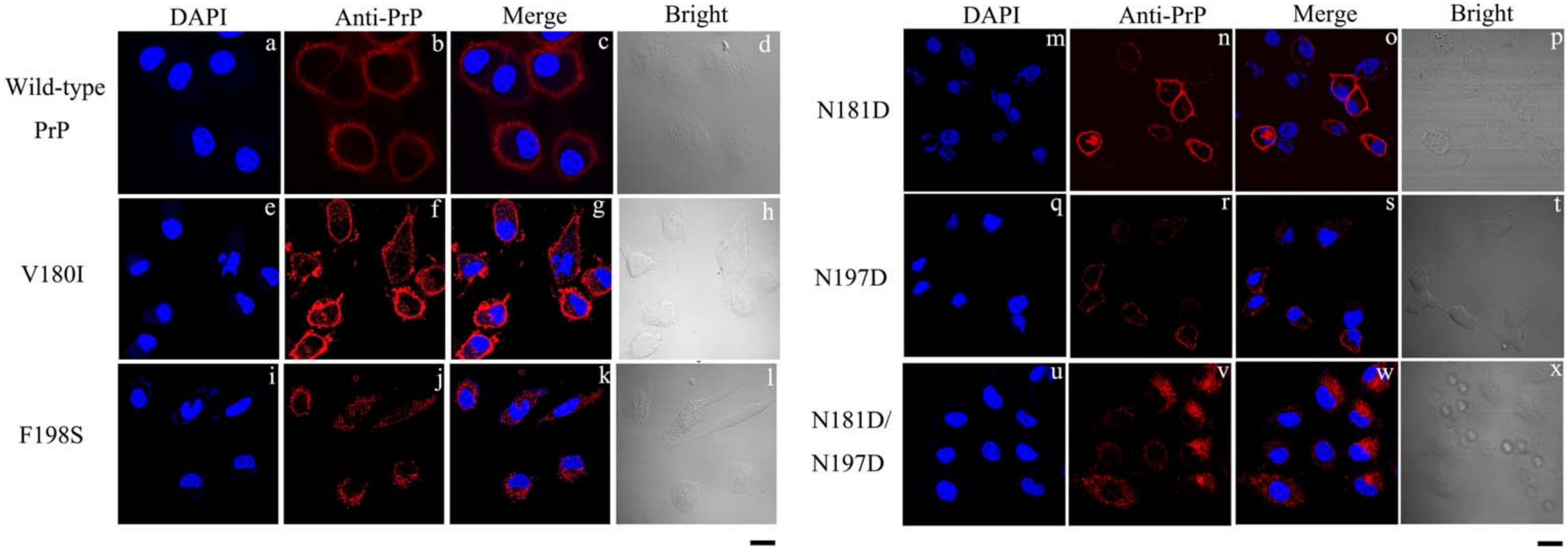
DAPI
Anti-PrP
Merge
Bright
Wild-type PrP
V180I
F198S
N181D
N197D
N181D/ N197D
a
b
c
d
e
f
g
h
i
j
k
l
m
n
o
p
q
r
s
t
u
v
w
x

Figure S2

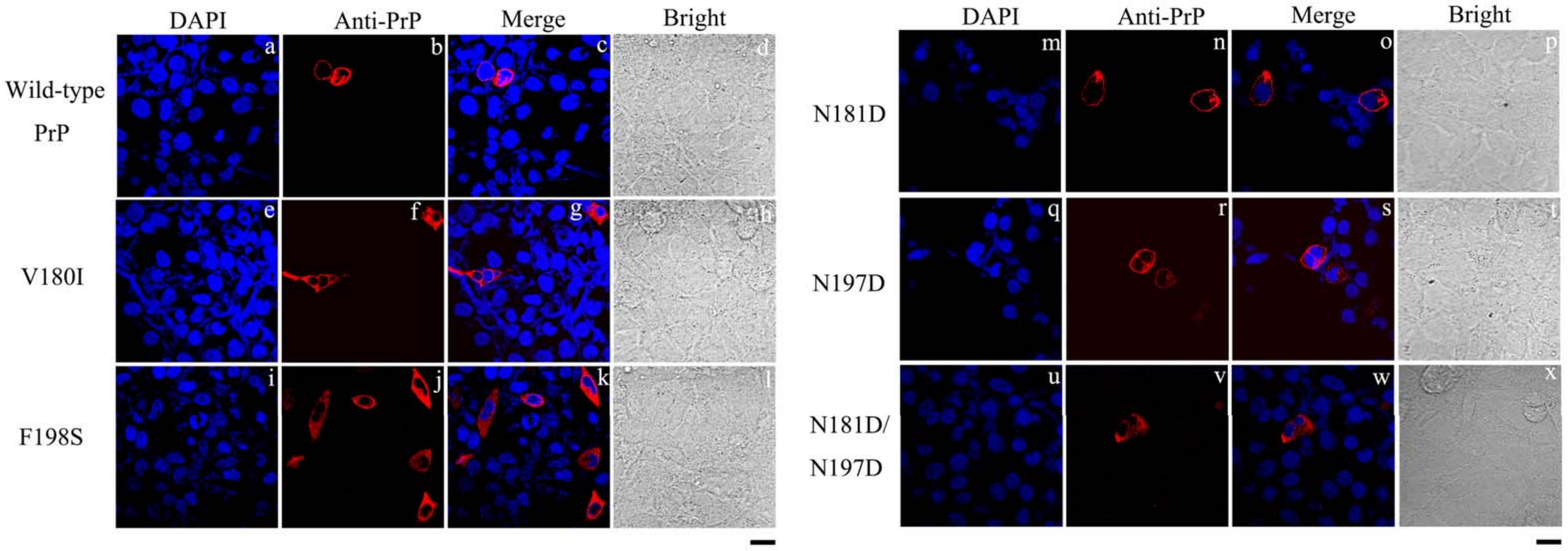
DAPI
Anti-PrP
Merge
Bright
Wild-type PrP
V180I
F198S
a
b
c
d
e
f
g
h
i
j
k
l
DAPI
Anti-PrP
Merge
Bright
N181D
N197D
N181D/ N197D
m
n
o
p
q
r
s
t
u
v
w
x

Figure S3

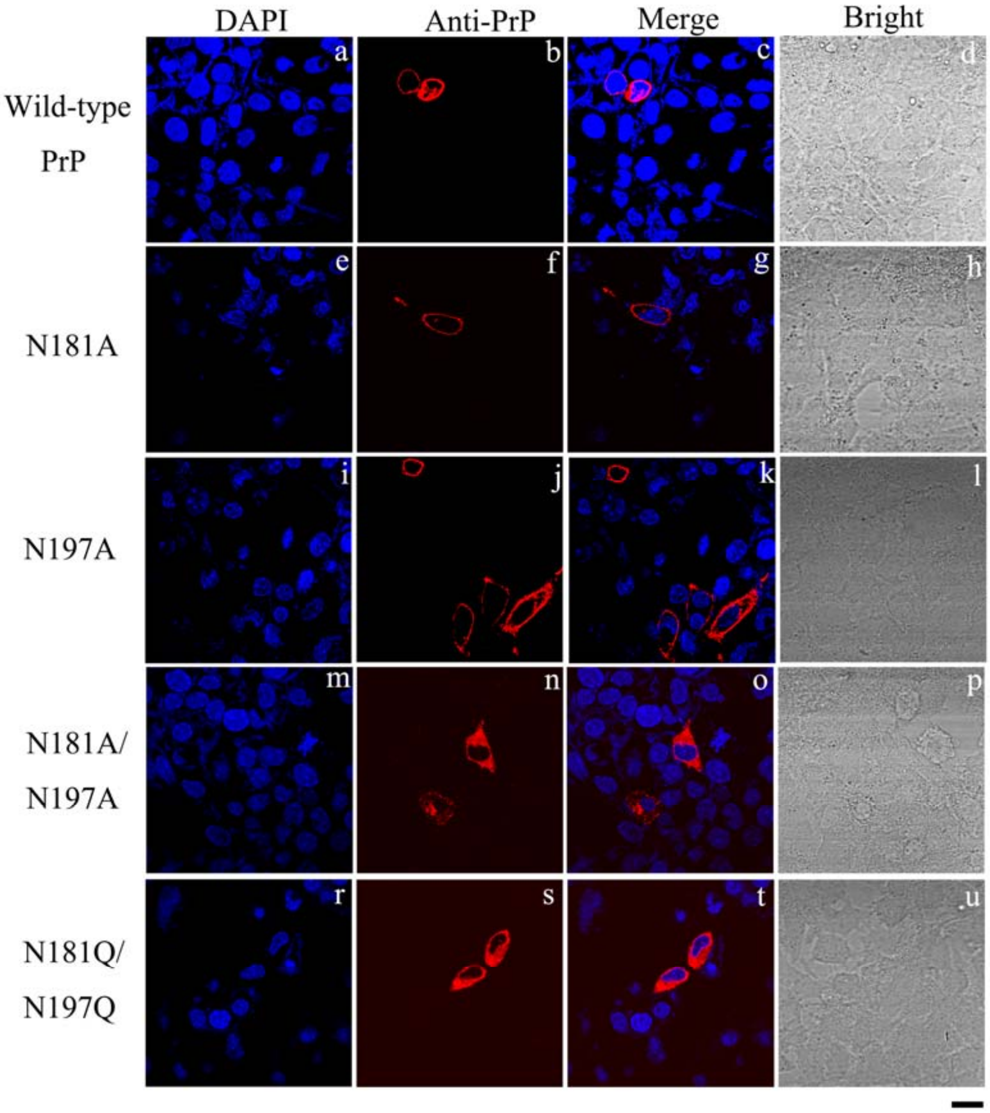
DAPI
Anti-PrP
Merge
Bright
Wild-type PrP
N181A
N197A
N181A/ N197A
N181Q/ N197Q
a
b
c
d
e
f
g
h
i
j
k
l
m
n
o
p
r
s
t
u

Figure S4a

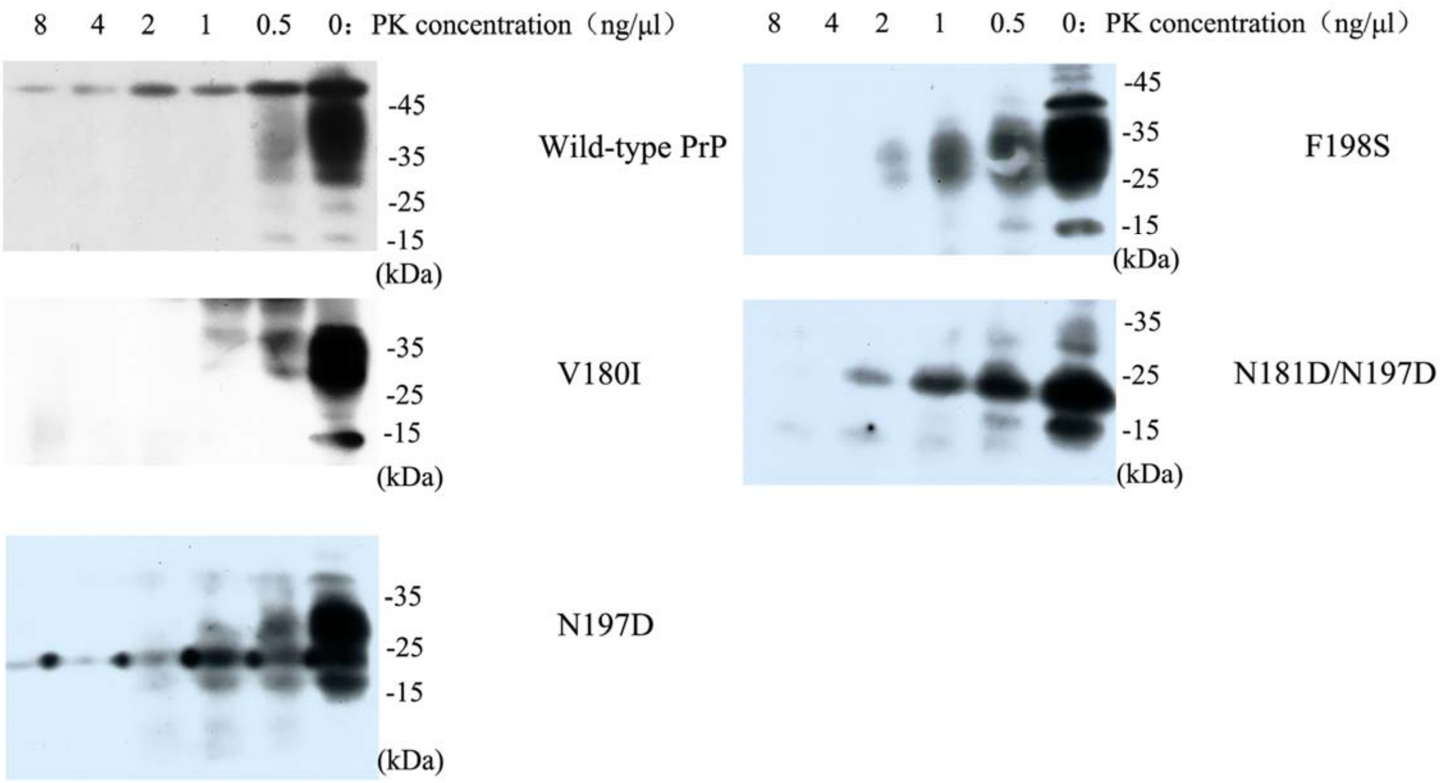

Figure S4b

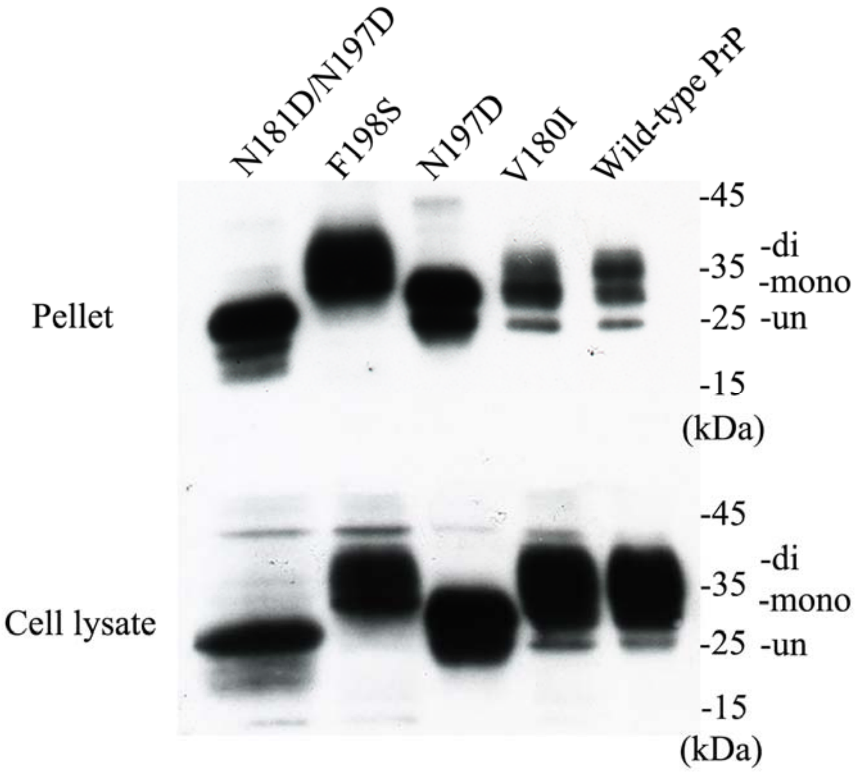

Figure S5a

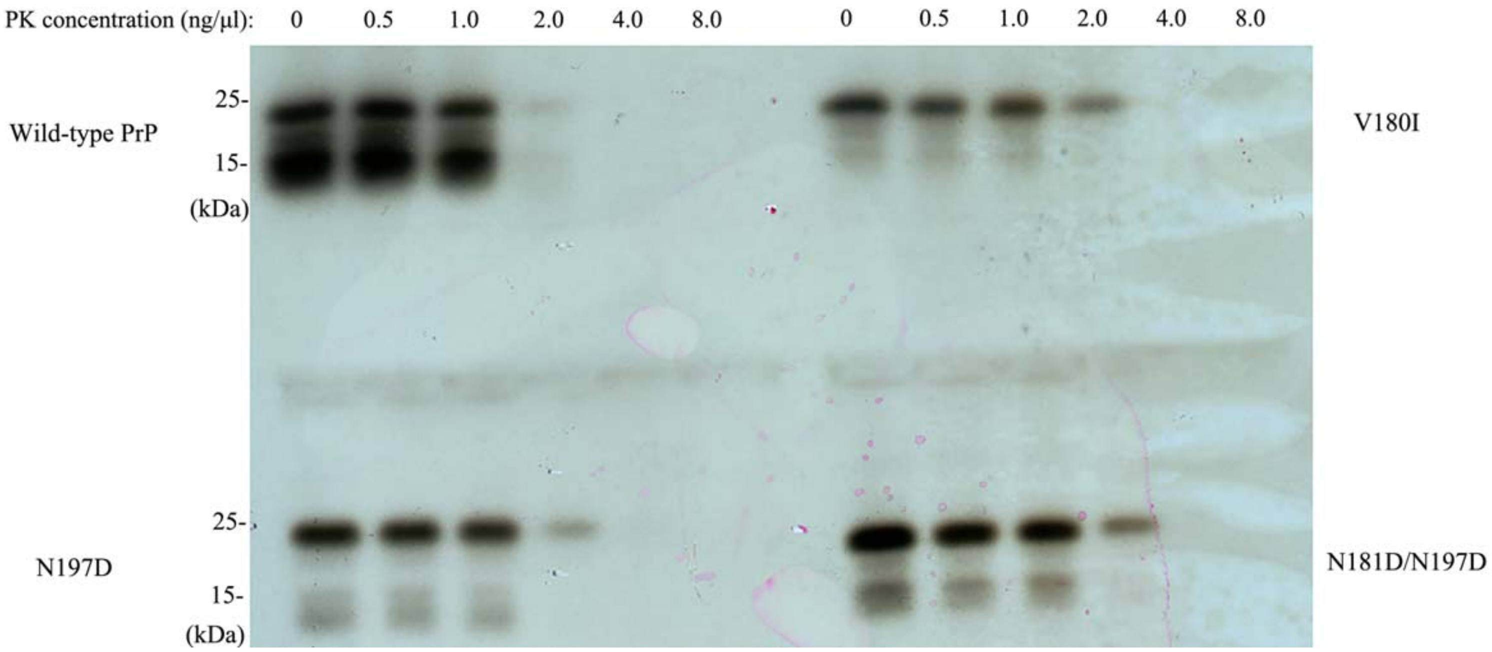

Figure S5b

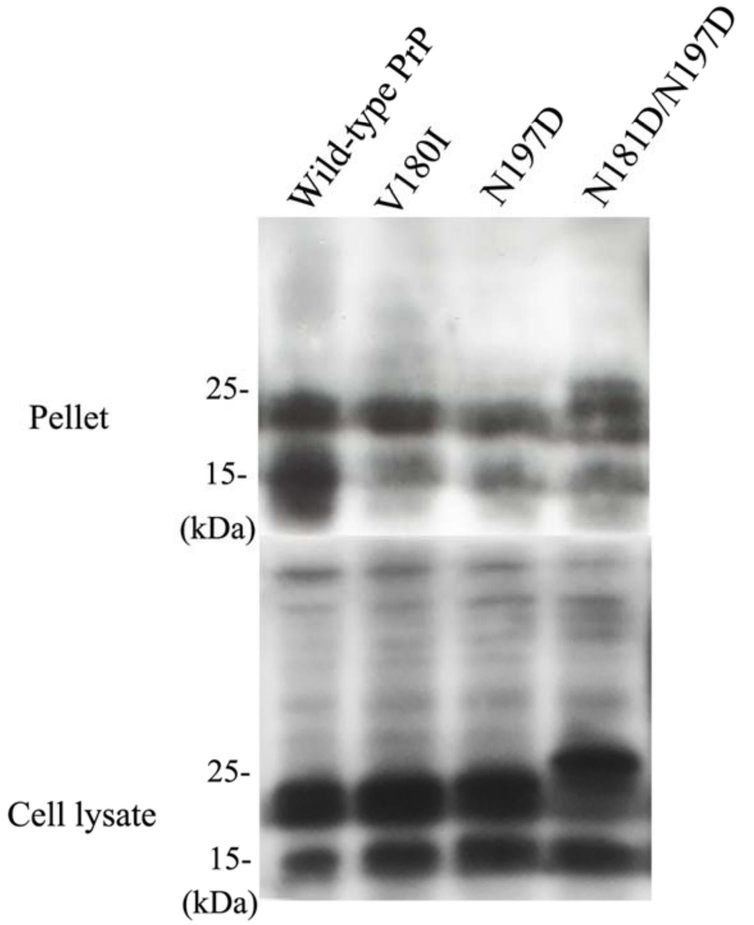

Figure S6

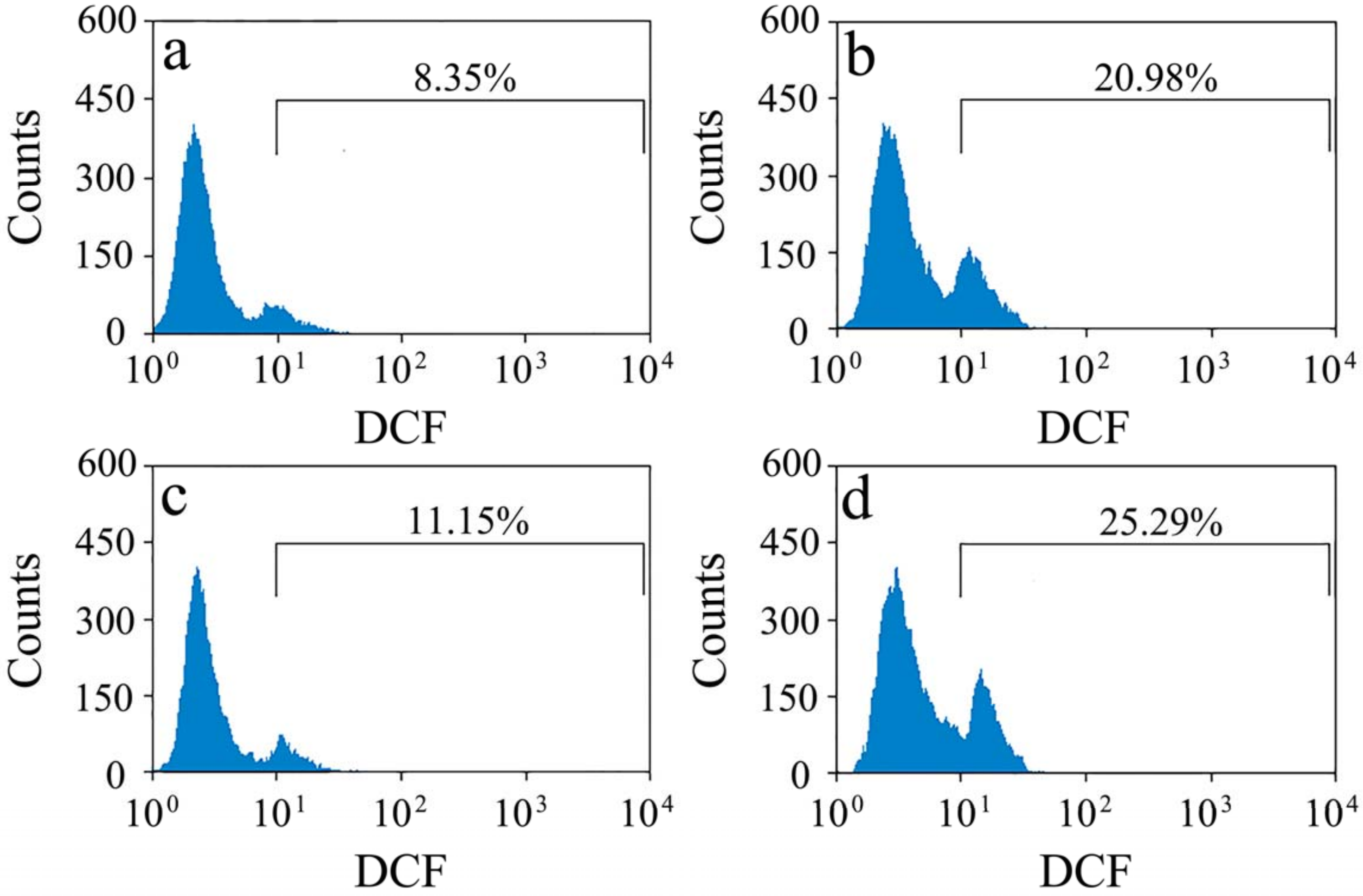
a
8.35%
b
20.98%
c
11.15%
d
25.29%
Counts
DCF
600
450
300
150
0
10^0
10^1
10^2
10^3
10^4

Figure S7

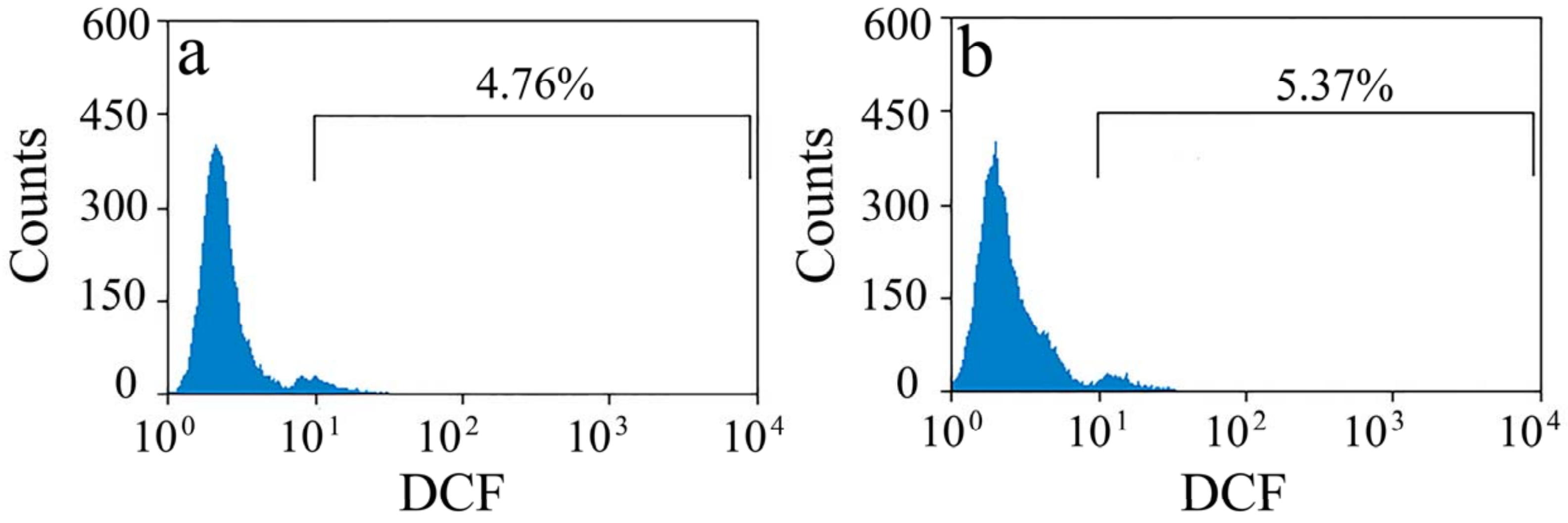
a
4.76%
Counts
600
450
300
150
0
10^0
10^1
10^2
10^3
10^4
DCF
b
5.37%
Counts
600
450
300
150
0
10^0
10^1
10^2
10^3
10^4
DCF

Figure S8

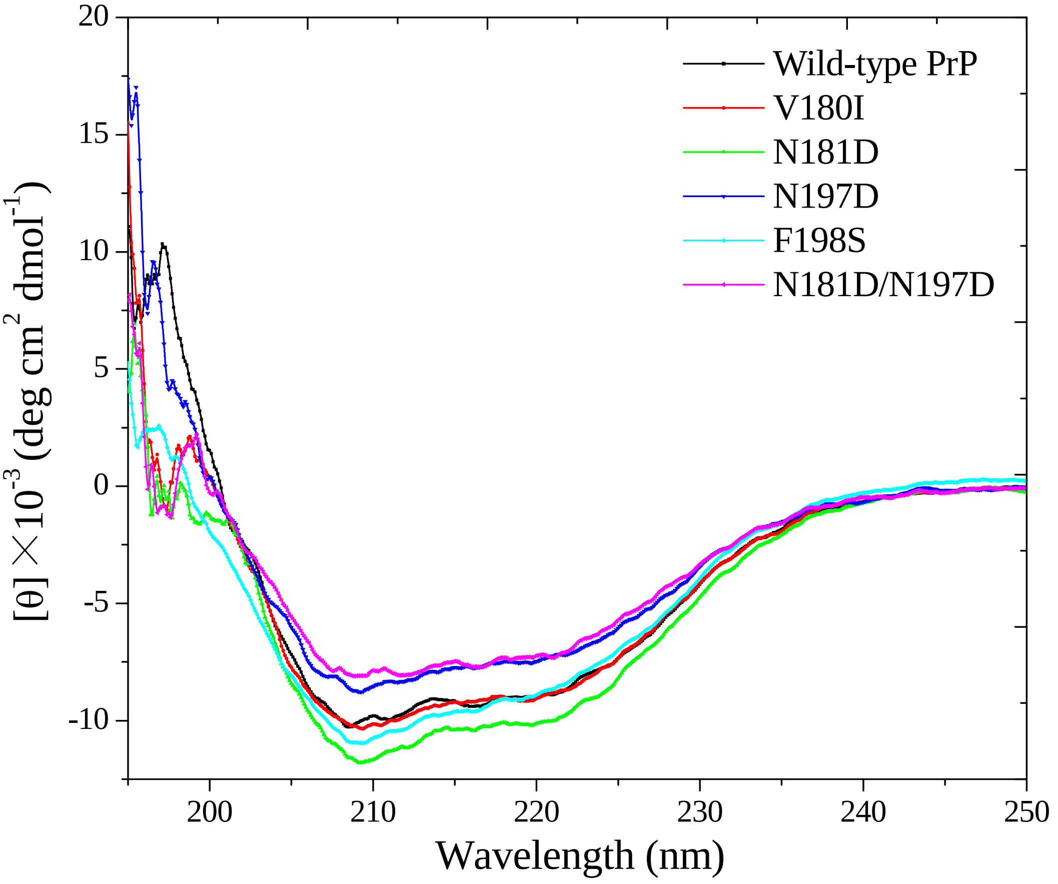

Wild-type PrP
V180I
N181D
N197D
F198S
N181D/N197D
20
15
10
5
0
-5
-10
200
210
220
230
240
250
Wavelength (nm)
[θ] ×10^-3 (deg cm^2 dmol^-1)

Figure S9

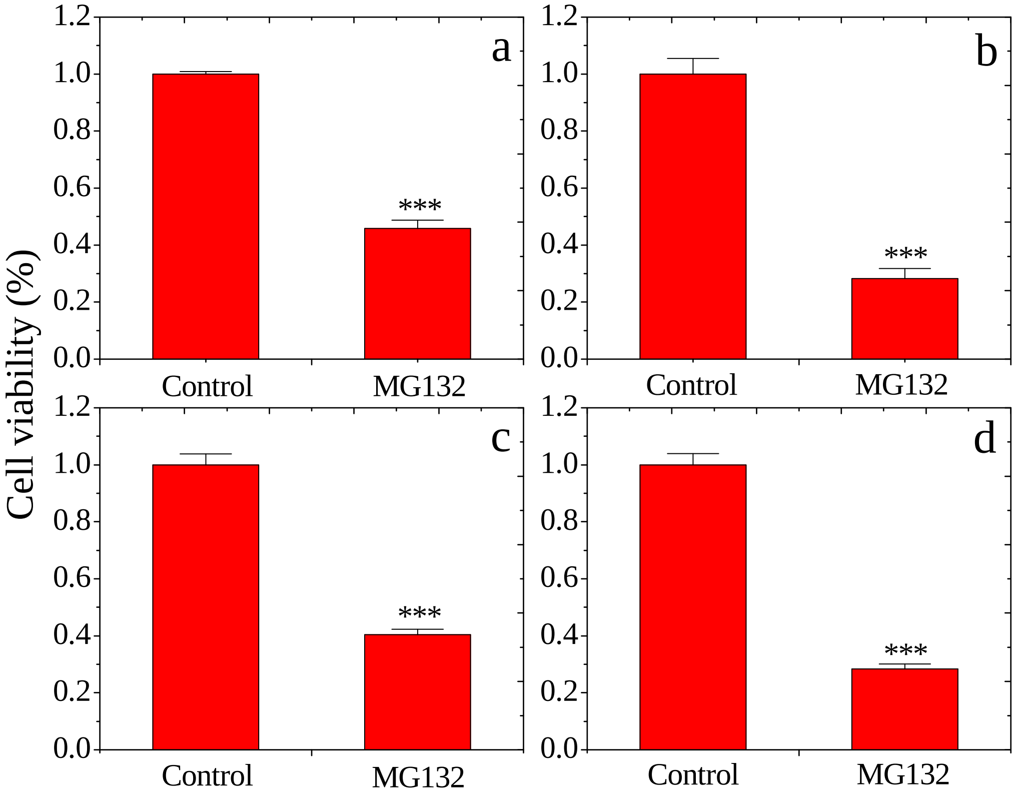

Figure S10

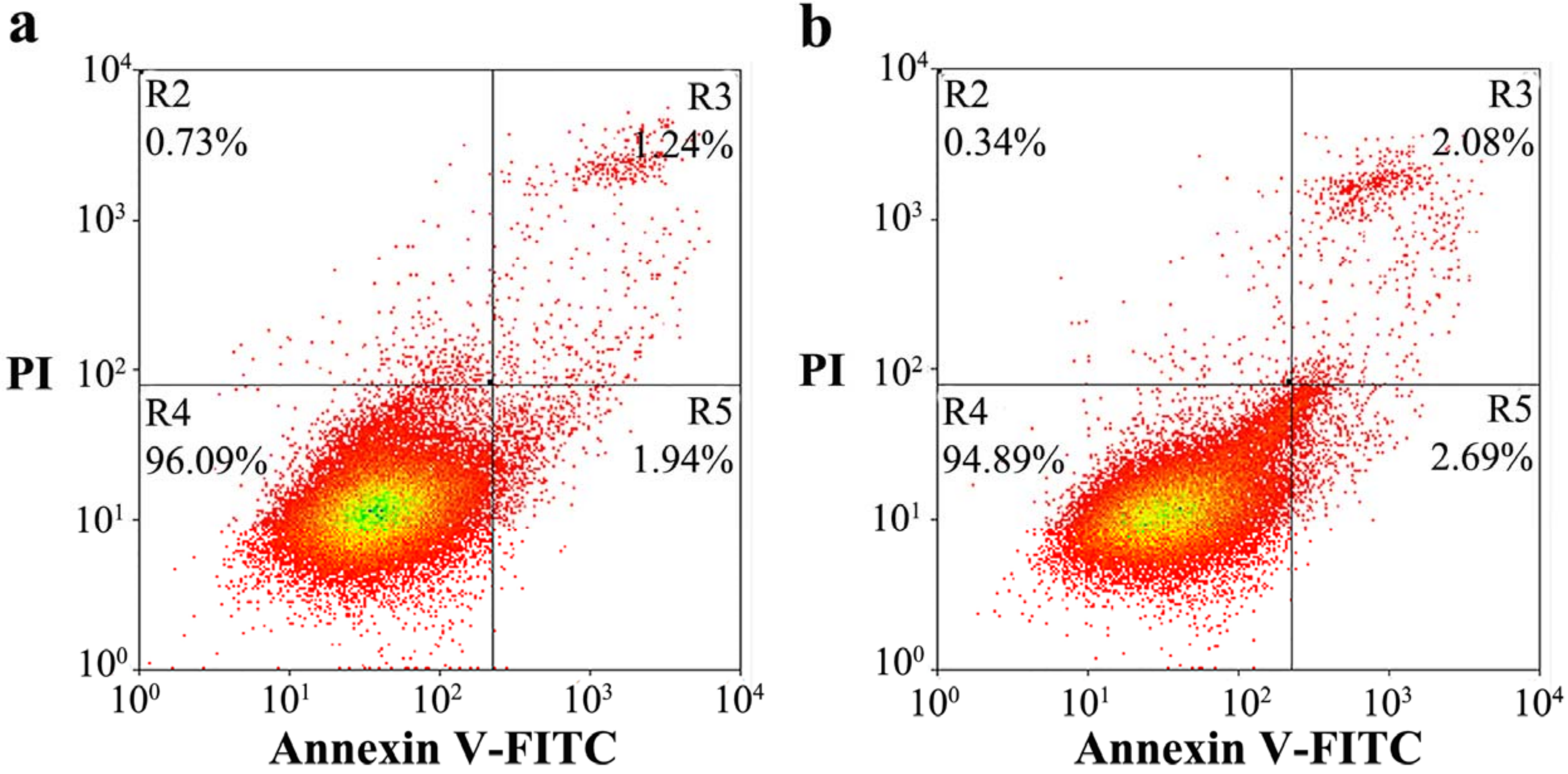
a
R2
0.73%
R3
1.24%
R4
96.09%
R5
1.94%
PI
Annexin V-FITC
b
R2
0.34%
R3
2.08%
R4
94.89%
R5
2.69%
PI
Annexin V-FITC

Figure S11

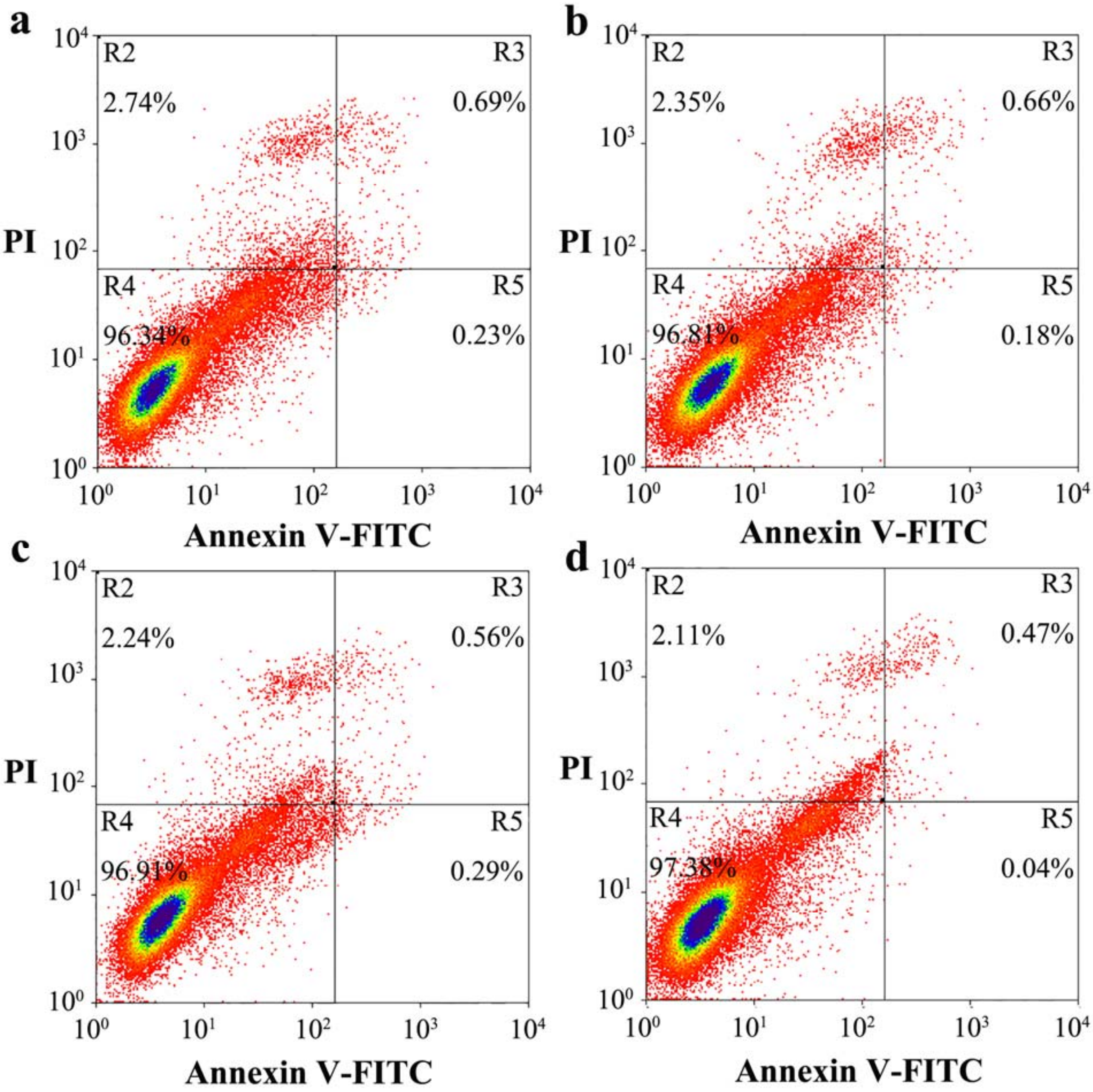
a
b
c
d
R2
R3
R4
R5
2.74%
0.69%
96.34%
0.23%
2.35%
0.66%
96.81%
0.18%
2.24%
0.56%
96.91%
0.29%
2.11%
0.47%
97.38%
0.04%
PI
Annexin V-FITC

Figure S12

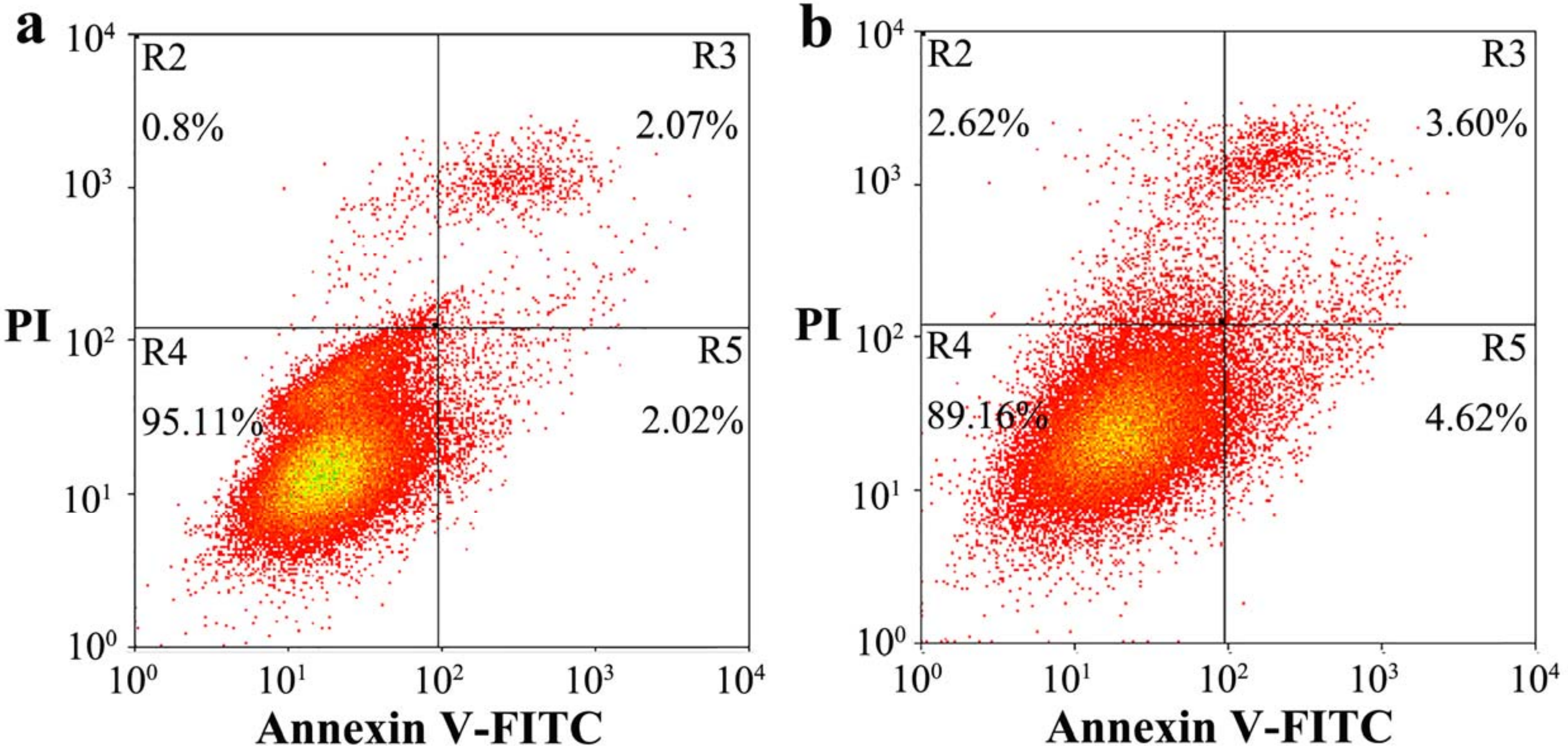
a
R2
0.8%
R3
2.07%
R4
95.11%
R5
2.02%
PI
Annexin V-FITC
b
R2
2.62%
R3
3.60%
R4
89.16%
R5
4.62%
PI
Annexin V-FITC

Figure S13

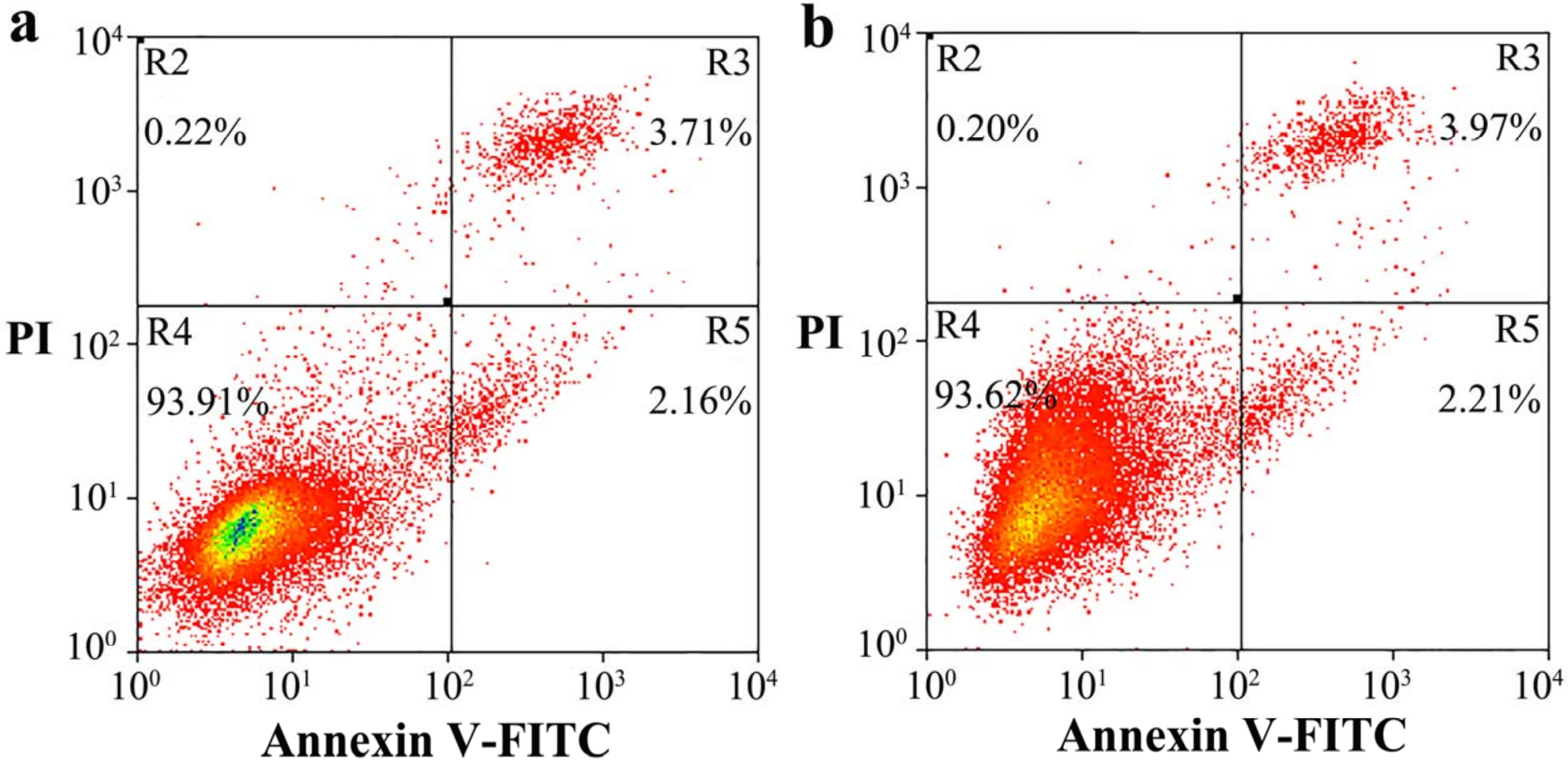
a
R2
0.22%
R3
3.71%
R4
93.91%
R5
2.16%
PI
Annexin V-FITC
b
R2
0.20%
R3
3.97%
R4
93.62%
R5
2.21%
PI
Annexin V-FITC
